# A Bcl-2 Associated Athanogene (*bagA*) Modulates Sexual Development and Secondary Metabolism in the Filamentous Fungus *Aspergillus nidulans*

**DOI:** 10.3389/fmicb.2018.01316

**Published:** 2018-06-15

**Authors:** Sachin Jain, Philipp Wiemann, Elizabeth Thill, Brett Williams, Nancy P. Keller, Mehdi Kabbage

**Affiliations:** ^1^Department of Plant Pathology, University of Wisconsin-Madison, Madison, WI, United States; ^2^Department of Medical Microbiology and Immunology, University of Wisconsin-Madison, Madison, WI, United States; ^3^Centre for Tropical Crops and Biocommodities, Queensland University of Technology, Brisbane, QLD, Australia; ^4^Department of Bacteriology, University of Wisconsin-Madison, Madison, WI, United States

**Keywords:** Bcl-2 associated athanogene, sexual development, *Aspergillus*, secondary metabolism, development, B-cell lymphoma 2 (Bcl-2) family, reproduction

## Abstract

The Bcl-2 associated athanogene (Bag) family is a multifunctional group of proteins distinguished by a conserved region known as the Bag domain (BD). Herein, we discuss the discovery and characterization of a Bag protein in the model genetic fungus *Aspergillus nidulans*, we designated BagA. BagA shares striking similarities in 3D structure, domain organization, amino acid properties, and Hsp70 binding surfaces to animal and plant Bags. While Hsp70 binding is a common feature of Bag proteins, our experimental evidence shows that BagA does not cooperate with *A. nidulans* Hsp70s, suggesting this association may not be a universal feature of Bag proteins. Gene expression of *bagA* was strongly induced during sexual development suggesting a role in developmental processes. Accordingly, the deletion of *bagA* (Δ*bagA*) negatively impacted sexual development, while its overexpression resulted in constitutive induction of sexual fruiting bodies and spores. Asexual and sexual development was linked to secondary metabolism in *A. nidulans*. Our data show that the deletion of *bagA* also provoked an altered secondary metabolite (SM) profile in both sexual and vegetative growth phases. Indeed, LC-MS analysis showed a significant enrichment of SMs in Δ*bagA*, including novel metabolites not produced by wild type strain. Enrichment of SMs in Δ*bagA* strain is particularly intriguing and suggest that altering cellular homeostasis can be used as a provocative strategy to activate cryptic metabolites and uncover novel bioactive compounds. Overall, our results indicate that Bag proteins in filamentous fungi share developmental regulatory roles with their animal and plant counterparts. We also show a potentially unique role for BagA in modulating secondary metabolism in *A. nidulans*. To our knowledge, this study provides a first insight into Bag function in filamentous fungi.

## Introduction

The Bcl-2 associated athanogene (Bag) family is a multifunctional, evolutionarily conserved group of proteins with homologs found across large evolutionary distances, ranging from yeast to humans (Knee et al., 2001; Kabbage and Dickman, 2008; Kabbage et al., 2017). The first Bag protein was discovered in an interactor screen using the human B cell lymphoma 2 (Bcl-2) as bait, and was termed Bag1, for Bcl-2 associated athanogene 1. Bag1 was implicated in apoptotic pathways and was found to enhance the anti-apoptotic activity of Bcl-2 (Knee et al., 2001). All Bag proteins contain a conserved C-terminal motif, termed the Bag domain (BD), which largely mediates interaction with the ATPase domain of the Hsp70 molecular chaperones (Knee et al., 2001; Kabbage and Dickman, 2008). Further studies have described Bag proteins as co-chaperones that function as molecular switches acting upon multiple targets to maintain protein homeostasis (Behl, 2016).

Much of our understanding of Bag function comes from studies in mammalian cells and the model plant *Arabidopsis thaliana*. The human genome encodes six Bag members (Bag1-6), all of which contain the signature BD (Knee et al., 2001; Kabbage and Dickman, 2008). Bag1, the founding member of this family, also contains a ubiquitin-like (UBL) domain, suggesting a role in protein degradation via the proteasome system. In addition to its interaction with Bcl-2, BAG1 has also been implicated in multiple signaling events through its association with hormone receptors, p53-mediated apoptotic events, and Raf1 protein kinase (Nollen and Morimoto, 2002). Bag2, 3, and 4 were identified as Hsp70 binding partners, and were shown to regulate its function (Takayama et al., 1999). Recently, Bag3 was discussed as a potential target in cancer therapies due to its maintenance of tumor cell survival during chemotherapy (Rosati et al., 2007). Bag4 is proposed to maintain survival by negatively regulating death receptors, such as, TNF1 and death receptor 3 (Jiang et al., 1999). The last two members; Bag5 and 6, were implicated in neurodegenerative disorders (Beilina et al., 2014) and developmental processes (Desmots et al., 2005), respectively. In *Arabidopsis*, seven Bag homologs were identified based on structural conservation of the BD (Doukhanina et al., 2006). Based on their domain organization, *Arabidopsis* Bags can be divided into two groups. AtBag1-4 are structurally similar to the human Bag1 and contain both BD and UBL domains. AtBAG5-7 contain a calmodulin (CaM)–binding domain near the BD, a unique feature associated with plant Bag proteins. Overall, plant Bags appear to function mostly in cytoprotection, in response to developmental and stress cues, similarly to animal Bags (Doukhanina et al., 2006; Williams et al., 2010; Li Y. et al., 2016; Kabbage et al., 2017; Li et al., 2017). These plant and human studies suggest a conserved and critical role of Bag proteins in a remarkable array of cellular functions. In *Saccharomyces cerevisiae*, a Bag-domain containing protein, SNL1, was identified and shown to interact with yeast Hsp70 as well as associate with ribosomes. Null mutants of SNL1 did not seem to have any detectable phenotypes and SNL1 was proposed to function as a recruiter of Hsp70 and support protein biogenesis via ribosome association (Sondermann et al., 2002; Verghese and Morano, 2012). Despite the conservation of Bag proteins from yeast to humans, their function in filamentous fungi is currently unknown. In this study, we identify and investigate the role of a Bag protein in the model fungal organism *Aspergillus nidulans*.

*Aspergillus nidulans* is a homothallic filamentous fungus with distinct developmental stages including vegetative, asexual, and sexual life stage. These developmental stages are genetically controlled by a series of programmed events including genetic factors and environmental cues including light, pH, and temperature (Mooney and Yager, 1990; Tilburn et al., 1995; Purschwitz et al., 2008). In *A. nidulans*, light plays an essential role in inducing asexual sporulation (conidia) whereas in absence of light, fungus initiates formation of sexual fruiting bodies (cleistothecia) containing sexual spores (ascospores). Some of the major players involved in sexual and asexual developmental balance are well known and characterized including velvet complex members (VeA, VelB, and LaeA) (Bayram et al., 2008) and fungal dioxygenases (Ppo proteins) (Tsitsigiannis et al., 2004, 2005). Despite this knowledge, sexual and asexual development is multifactorial and continues to be an active area of investigation. In addition to genetic factors and environmental cues, many secondary metabolites influence both asexual (Hicks et al., 1997) and sexual development (Bayram et al., 2008; Bayram and Braus, 2012) in fungi.

Using pfam domain-based and hidden Markov models (HMMs), we identified a Bag homolog in *A. nidulans* (AN12105) that we named BagA (Bcl-2 associated athanogene A). Unlike its human and plant counterparts, *A. nidulans* contain a single *bag* gene, as it is the case of many other fungal organisms. Similar to human Bag1 and AtBag1-4, BagA contains a conserved C-terminal BD and a UBL domain at its N-terminus. Here we show that BagA affects sexual development and secondary metabolite output of *A. nidulans*. Deletion of *bagA* reduced sexual development and a significant increase in the accumulation of secondary metabolites. Conversely, overexpression significantly increased the production of sexual fruiting bodies and sexual spores. In addition to the aforementioned role of Bag proteins in animals and plants, altering SM output appears to be a novel role for *A. nidulans* BagA, which suggests a functional divergence of fungal Bags. Overall, our results indicate that the developmental aspects of Bag regulation are conserved in filamentous fungi, and implicate Bag in both sexual and asexual reproduction, and secondary metabolism in *A. nidulans*.

## Materials and methods

### Fungal strains and culture conditions

The strains and plasmids used in this study are listed in Table S1. All strains were grown at either 37 or 30°C and maintained as glycerol stocks at −80°C. *In silico* analysis was done using Fungidb (http://fungidb.org/fungidb/), and NCBI (https://www.ncbi.nlm.nih.gov/). *In silico* homology modeling was done using Phyre2 web portal (http://www.sbg.bio.ic.ac.uk/phyre2/html/page.cgi?id=index) and Pymol software was used to visualize 3D structures of the protein.

### Physiological studies

Strains were assessed for sexual and asexual development by quantification of ascospores or conidia, respectively. Briefly, 10^6^ Conidia per ml were inoculated in 0.75% molten agar and overlaid on a 1.5% solid glucose minimal media (GMM) (Shimizu and Keller, 2001) and incubated in dark for 7 days to induce sexual development whereas white light illumination was used to induce asexual sporulation for 6 days. Cultures were grown in continuous light or dark for the specified time. At respective time intervals, 12.5 mm diameter cores were taken and homogenized in 3 ml of water to release spores. Serial dilution was performed, and spores were counted using hemocytometer. All experiments were done in triplicates and statistical analysis was done using Two-way ANNOVA test in Graphpad® prism software.

### Northern analysis

The strains were analyzed for gene expression using standard Northern blotting procedure (Sambrook and, 2001). 10^6^ spores/ml were grown in liquid minimal media for 24 h at 37°C shaking at 250 rpm. Total RNA was extracted using Qiazol Reagent (Qiagen) as described previously (Sambrook and, 2001). Approximately 15 μg of total RNA was run on formaldehyde agarose gel and transferred to nitrocellulose membrane for blotting. Blots were hybridized with DNA fragments generated by PCR of wild-type (WT) genomic DNA with gene specific primers.

### Transformation and genetic manipulation

Fungal transformation was performed as previously described (Palmer et al., 2008). Construct for the overexpression of *bag* was made using double-joint PCR (Yu et al., 2004; Szewczyk et al., 2006). Briefly, approximately 1.5-kb 5′ flanking region of *bagA* was PCR amplified from genomic DNA (gDNA) of RJMP103.5 and fused to the *A. parasiticus pyrG::gpdA (P)* from plasmid pJMP9 and the *bag* open reading frame (primers are listed in Table S2). Over expression of *bag* was achieved by transformation of the construct into TNO2A7 (*pyrG1, pyro1*, and *ribo1*), which created TSJA12.1. These strains were confirmed by Southern blot. Deletion of *bag* was achieved by using double joint PCR (Yu et al., 2004; Szewczyk et al., 2006). Approximately 1 Kb of the 5′ and 3′ flanking region was PCR amplified from gDNA of RJMP 103.5 and fused to *A. parasiticus pyrG* gene from pJW24. The deletion cassette was transformed into TNO2A7 (*pyrG1, pyro1*, and *ribo1*) to generate TSJA13.1 which was confirmed by southern blotting as well. RJMP101.19 (*pyrG*89, *veA*+) was used for sexual crosses. TSJA12.1 and TSJA13.1 were crossed separately with RJMP101.19 to generate *veA*+ prototrophic strains RSJA6.1 (OE::*bagA*) and RSJA7.1 (Δ*bagA*). TSJA13.1 was also crossed with TDIT5.8 strain to generate RSJA14.1 (OE::*ppoA*; Δ*bagA*) prototrophic strain. *bagA* complementation construct was generated using double joint PCR. Briefly, *bagA* (ORF, 1 kb of 5′ flank, and 500 bp of 3′ flanks) was fused to *A. fumigatus pyroA* (selection marker), approximately 1 kb 5′, and 3′ flanks of *A. nidulans pyrG*. Fusion PCR product was then transformed into TSJA13.1 by complementing *bagA* at the *pyrG* locus generating Bag-Full-C strain. Similarly, mutated D368S *bagA* was fused to *pyroA* and *pyrG* to generate D368S strain. Mutation was confirmed by sequencing. BagA::Flag strain was generated using plasmid pHS13 containing a 3xFLAG tag and the trpC terminator (Park et al., 2012). Double joint PCR was utilized to fuse the C-terminus of BagA with 3xFLAG and trpC terminator amplified from pHS13 using PyrG as selectable marker amplified from pJW24. PCR product was transformed into TNO2A7 and crossed back to RJMP 101.19 to generate prototrophic strain RSJA 18.1. All the strains were confirmed with PCR, southern, and northern blotting as appropriate. All assays were performed using RSJA6.1 (OE::*bagA*) and RSJA7.1 (Δ*bagA*).

### Yeast-two-hybrid assay

A directed yeast-two-hybrid system was used to test BagA and Hsp70 association based on the Matchmaker Gold Yeast Two-Hybrid (Clontech). A PCR product was obtained using *bagA* gene specific primers and subsequently cloned into the bait vector pGBKT7 and confirmed by sequencing. Similarly, Prey vectors containing Hsp70 ORFs were constructed in pGADT7. Bait and prey plasmids were then transformed into *Saccharomyces cerevisiae* AH109 host according to manufacturer's instructions, and protein-protein interactions were assayed by plating on synthetic drop-out media lacking Leucine (Leu), Tryptophan (Trp), and Histidine (His).

#### Protein extraction and immunoprecipitation

For preparation of the protein samples from mycelium, conidia (5 × 10^5^ conidia/ml) of wild type and BagA::Flag strains were inoculated in 50 ml of liquid minimal media and incubated for 24 h at 37°C. Mycelial samples were collected, freeze dried and stored at −80°C. Prepared mycelial samples were resuspended in lysis buffer B250 (100 mM Tris-HCl, pH 7.5, 250 mM NaCl, 10% glycerol, 1 mM EDTA, and one Roche Complete Protease inhibitor tablet without EDTA per 50 ml) as previously described (Park et al., 2012; Patananan et al., 2013), broken by a mini-bead beater for 2 cycles (1 min homogenization with 10 min sitting on ice) and centrifuged in a microcentrifuge for 15 min at 15,000 rpm at 4°C. The supernatant was incubated with anti-FLAG M2 magnetic beads and elution was performed according to manufacturer's protocol (M8823, Sigma). Total elution fractions were sent for sequencing and further processing.

#### Enzymatic “in liquid” digestion

Immunoprecipitated protein samples were TCA/acetone precipitated [10% TCA, 28% acetone final] then pellets re-solubilized and denatured in 7.5 μl of 8M Urea/50 mM NH_4_HCO_3_ (pH8.5)/1 mM TrisHCl for 10 min. Subsequently diluted to 30 μl for reduction step with: 1.25 μl of 25 mM DTT, 2.5 μl MeOH, 18.75 μl 25 mM NH_4_HCO_3_ (pH8.5). Incubated at 52°C for 15 min, cooled on ice to room temperature then 1.5 μl of 55 mM IAA was added for alkylation and incubated in darkness at room temperature for 15 min. Reaction was quenched by adding 4 μl of 25 mM DTT. Subsequently 1 μl of Trypsin/LysC solution [100 ng/μl *Trypsin/LysC* mix in 25 mM NH_4_HCO_3_] and 13.5 μl of 25 mM NH_4_HCO_3_ (pH8.5) was added to 50 μl final volume. Digestion was conducted for 2 h at 42°C then additional 0.5 μl of trypsin/LysC solution added and digestion proceeded o/n at 37°C. Reaction was terminated by acidification with 2.5% TFA [Trifluoroacetic Acid] to 0.3% final.

#### Nano LC-MS/MS

Digest was cleaned up using OMIX C18 SPE cartridges (Agilent, Palo Alto, CA) per manufacturer protocol and eluted in 10 μl of 70/30/0.1% ACN/H_2_O/TFA, dried to completion in the speed-vac and finally reconstituted in 12 μl of 0.1% formic acid. connected to a hybrid linear ion trap-orbitrap mass spectrometer (LTQ-Orbitrap Elite™, Thermo Fisher Scientific) equipped with an EASY-Spray™ electrospray source. Chromatography of peptides prior to mass spectral analysis was accomplished using capillary emitter column (PepMap® C18, 3 μM, 100 Å, 150 × 0.075 mm, Thermo Fisher Scientific) onto which 3 μl of extracted peptides was automatically loaded. NanoHPLC system delivered solvents A: 0.1% (v/v) formic acid, and B: 99.9% (v/v) acetonitrile, 0.1% (v/v) formic acid at 0.50 μL/min to load the peptides (over a 30 min period) and 0.2 μl/min to elute peptides directly into the nano-electrospray with gradual gradient from 3% (v/v) B to 30% (v/v) B over 77 min and concluded with 5 min fast gradient from 30% (v/v) B to 50% (v/v) B at which time a 5 min flash-out from 50 to 95% (v/v) B took place. As peptides eluted from the HPLC-column/electrospray source survey MS scans were acquired in the Orbitrap with a resolution of 120,000 followed by MS2 fragmentation of 20 most intense peptides detected in the MS1 scan from 350 to 1,800 m/z; redundancy was limited by dynamic exclusion.

#### Peptide data analysis

Raw MS/MS data were converted to mgf file format using MSConvert (ProteoWizard: Open Source Software for Rapid Proteomics Tools Development) for downstream analysis. Resulting mgf files were used to search against *Aspergillus nidulans* amino acid sequence database containing a list of common contaminants (21,187 total entries) using in-house *Mascot* search engine 2.2.07 [Matrix Science] with variable Methionine oxidation with Asparagine and Glutamine deamidation plus fixed cysteine Carbamidomethylation. Peptide mass tolerance was set at 15 ppm and fragment mass at 0.6 Da. Protein annotations, significance of identification and spectral based quantification was done with help of Scaffold software (version 4.3.2, Proteome Software Inc., Portland, OR). Protein identifications were accepted if they could be established at greater than 80.0% probability within 1% False Discovery Rate and contained at least 2 identified peptides. Protein probabilities were assigned by the Protein Prophet algorithm (Nesvizhskii et al., 2003). Proteins that contained similar peptides and could not be differentiated based on MS/MS analysis alone were grouped to satisfy the principles of parsimony.

#### Untargeted metabolomics using LC/MS

Two growth conditions were tested for total secondary metabolite profiles:

*Sexual development:* 10^6^ Conidia per ml were inoculated in 0.75% molten agar and overlaid on a 1.5% rich nutrient agar media and incubated in dark for 7 days to induce sexual development. 3 plates per strain were harvested using sterile glass slides into 15 ml round bottom tubes. Fungal tissue was homogenized in 5 ml of 50% acetonitrile. Samples were centrifuged at 4,000 rpm for 15 mins and supernatant was collected in different tubes. The supernatant was then passed through 0.2 μ filters and analyzed on UHPLC as described below.

*Vegetative development*: 10^6^ Conidia per ml were inoculated into 125 ml flasks containing 50 ml liquid minimal media. Strains were grown at 30°C for 5 days shaking at 250 rpm. Five-day old cultures were harvested, mycelia were filtered, and 5 ml of culture supernatant was extracted with 5 mls of organic solvent (Ethyl acetate). Top layer of the ethyl acetate was then taken off and instantly dried in speed-vac. Dried extracts were resuspended in 100% ACN, filtered through 0.2 μ filters, and analyzed on UHPLC as described below.

The samples were analyzed by ultra-high-performance liquid chromatography (UHPLC) coupled with mass spectrometry (MS). The samples were separated on a ZORBAX Eclipse XDB-C18 column (Agilent, 2.1 × 150 mm with a 1.8 μM particle size using a binary gradient of 0.1% (v/v) formic acid (FA) as solvent A and 0.1% (v/v) FA in acetonitrile (ACN) as solvent B that was delivered by a Vanquish^TM^ UHPLC system (Thermo Scientific) with a flow rate of 0.2 mL/min. The binary gradient started with 20% B that was increased with a linear gradient to 100% B in 15 min followed by an isocratic step at 100% B for 5 min. Before every run, the system was equilibrated for 5 min at 20% B. The UHPLC system was coupled to a Q Exactive hybrid quadrupole Oritrap^TM^ MS (Thermo Scientific). For electrospray ionization, the ion voltage was set at 3.5 kV in positive mode or −3.5 kV for negative mode. Nitrogen was used as sheath gas at a flow rate of 45 and as sweep gas at a flow rate of 2. Data analysis was performed using Xcalibur^TM^ (ThermoFisher Scientific) and Maven (Melamud et al., 2010) software. For detection of differentially abundance of masses, R-based XCMS online program (https://xcmsonline.scripps.edu) was used as previously described (Smith et al., 2006). Identification of known *A. nidulans* compounds was achieved as previously described (Barkal et al., 2016).

## Results

### Identification of a single *bag* gene in *Aspergillus nidulans*

The Bag protein family is a divergent group of proteins with a conserved domain across taxa termed the Bag domain (BD). Despite this conservation, Bag sequence homology between distant species remains low. Previous studies comparing *Arabidopsis* and animal Bags reported low sequence identities (13–25%) and similarities (32–46%) between their BDs (Doukhanina et al., 2006). Accordingly, these sequences are often annotated as hypothetical or unknown proteins in most fungal databases. We used Hidden Markov Model-based protein domain profiling methods (Pfam) to identify BD-containing proteins in fungi (Finn et al., 2016). This method allows for detection of remote homologs in a more sensitive manner based on their unique domain identifiers. Using Pfam domain number for BD (PF02179), we identified several BD proteins in fungi (Table 1). Interestingly, the majority of fungal genomes contain a single Bag family member, unlike their animal and plant counterparts (Kabbage and Dickman, 2008).

**Table 1:** Predicted Bag proteins in fungal and fungal-like organisms obtained from FungiDB database.

**Phylum**	**Organism**	**Gene ID**	**Molecular weight (daltons)**	**Protein length (amino acid)**
Ascomycetes	*A. oryzae RIB40*	AO090009000475	42,201	385
	*A. terreus NIH2624*	ATEG_03020	117,624	1,061
	*A. fumigatus Af293*	Afu1g06080	42,568	393
	*B. cinerea B05.10*	BC1G_05107	33,306	299
	*C. albicans SC5314*	C1_10530W_A	23,488	199
	*F. graminearum PH-1*	FGRAMPH1_01G05059	33,986	305
	*F. oxysporum f. sp. lycopersici 4287*	FOXG_04441	34,188	305
	*H. capsulatum NAm1*	HCAG_00149	97,307	878
	*M. oryzae 70-15*	MGG_05448	39,690	351
	*T. marneffei ATCC 18224*	PMAA_024110	46,067	421
	*S. pombe*	SPBC16G5.11c	22,205	195
	*S. sclerotiorum 1980 UF-70*	SS1G_07244	33,117	297
	*S. cerevisiae S288c*	YIL016W	18,306	159
	*A. nidulans FGSC A4*	AN12105	44,491	401
Basidiomycetes	*P. graminis f. sp. tritici*	PGTG_10046	28,603	257
	*C. neoformans var. neoformans*	CNK01890	71,499	654
	*U. maydis 521*	UMAG_10984	29,547	269
	*M. globose CBS 7966*	MGL_3498	34,589	307
	*S. reilianum SRZ2*	sr15481	29,422	274
	*P. chrysosporium RP-78*	AGR57_12736	22,836	209
Zygomycetes	*M. circinelloides f. lusitanicus CBS 277.49*	QYA_155357	23,059	200
	*P. blakesleeanus NRRL 1555(-)*	PHYBL_59175	26,732	236
	*R. delemar RA 99-880*	RO3G_06850	48,792	409
Oomycetes	*P. ultimum DAOM BR144*	PYU1_G009272	40,214	351
	*P. infestans T30-4*	PITG_11572	41,969	363
	*H. arabidopsidis Emoy2*	HpaG808750	24,024	203
	*P. sojae strain P6497*	PHYSODRAFT_288977	43,146	378
	*S. diclina VS20*	SDRG_01156	37,480	336

A maximum-likelihood tree was constructed based on multiple sequence alignment of BDs and showed that Bag proteins are conserved in fungi and fungal-like organisms, and largely cluster according to their phylum designation (Figure S1). However, some fungal Bags appear more closely related to their counterparts in other kingdoms, indicating a close structural and possibly functional conservation across distant taxonomic groups (Figure S1). Due to low sequence similarities and miss-annotations in fungal databases, we speculate that many more fungal genomes have BD-containing proteins. In summary, this analysis highlights the evolutionary conservation of Bag proteins across wide evolutionary distances ranging from yeast to humans. In this study, we focus on a putative Bag protein in *Aspergillus nidulans* (AN12105) that we designated BagA following the *A. nidulans* nomenclature standards.

### BagA shares similar domain architecture as human Bag1 and *Arabidopsis* Bag1-4

**Figure 1:**
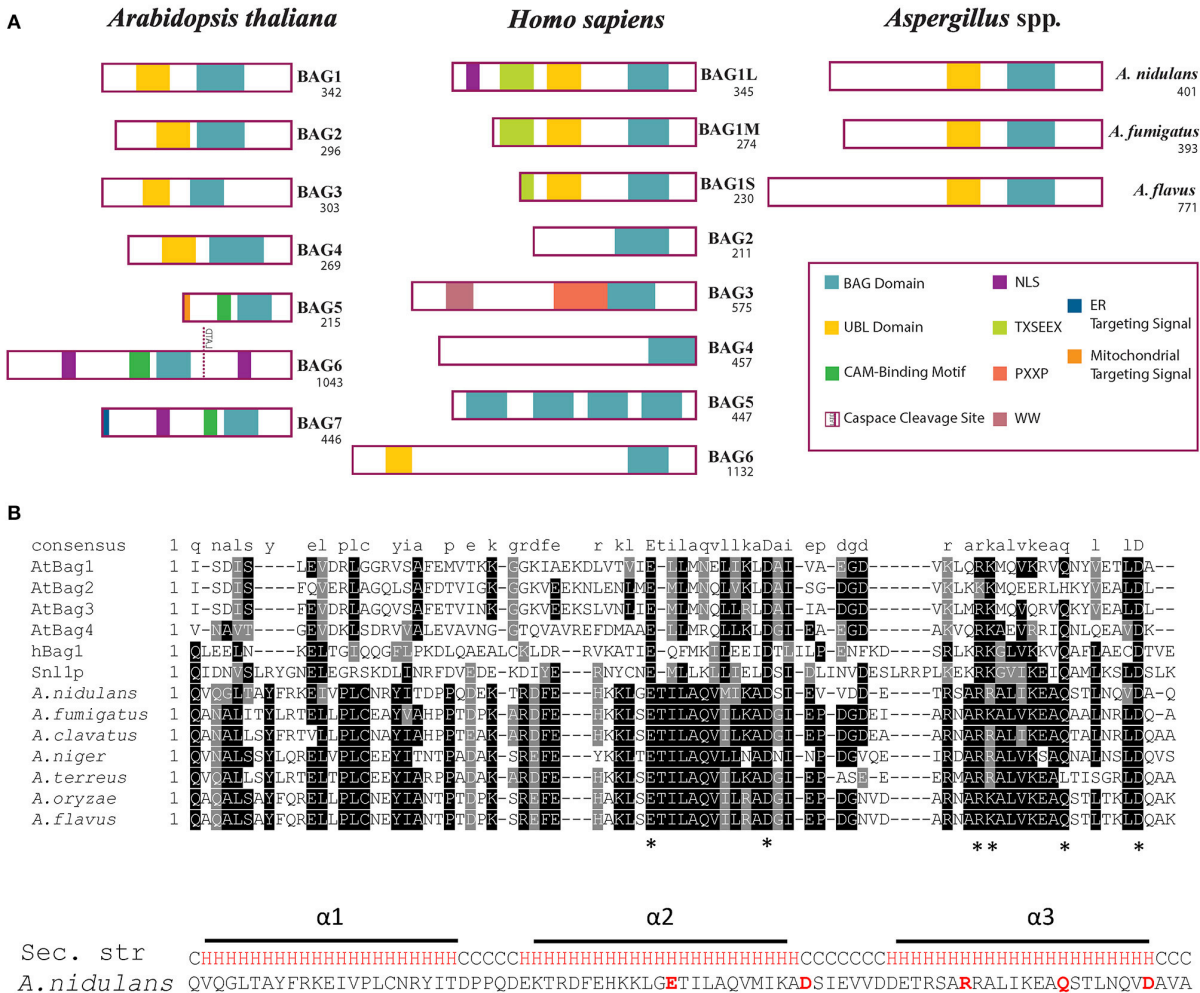
Domain architecture and multiple alignment of Bag proteins. **(A)** The domain archeture of *Arabidopsis thaliana* (At) Bag1-7, human (h) Bag1-6, and putative Bag proteins of *Aspergillus nidulans, A. fumigatus*, and *A. flavus* are compared. The number of amino acids for each protein are shown under the name. Abbreviations: Bag, Bcl-2 associated athanogene; UBL, Ubiquitin-like domain; CAM, Calmodulin; ER, Endoplasmic reticulum; NLS, Nuclear localization signal. **(B)** Bag domains of AtBag1-4, hBag1, *S. cerevisiae* Snl1, and *Aspergillus* spp. were aligned using ClustalW MSA program. The amino acid residues essential for Hsp70 binding are marked by asterisks “^*^”. The predicted secondary structure of *A. nidulans* BagA BD is shown with three alpha helices (H) highlighted in Red and coil (C) in black.

The domain architecture of BagA shares significant similarities with human Bag1 (hBag1) and *Arabidopsis* Bag1-4 (AtBag1-4) with a conserved BD at its C-terminus and an N-terminal ubiquitin-like (UBL) domain (Figure 1A). The BD is known to interact with the ATPase domain of Hsp70 molecular chaperones and regulates its function, the UBL domain is reported to communicate with the proteasome to assist in the removal of miss-aggregated proteins in mammals (Lüders et al., 2000). The BD region of BagA shared low sequence similarities to hBag1 and AtBag4 with similarity/identity percentages of 43/19 and 41/20, respectively. However, multiple sequence alignment of BDs of hBag1, AtBag1-4, and *Aspergillus* Bags revealed that key amino acid residues required for Hsp70 binding in humans and plants are conserved in *Aspergillus* spp. Functional and crystallography studies of the hBag1/Hsp70 complex revealed that residues Glu^212^, Asp^222^, Arg^237^, and Gln^245^ in hBag1 are essential Hsp70 binding (Briknarová et al., 2001). These key amino acid residues are conserved in *A. nidulans* BagA (Glu^357^, Asp^368^, Arg^381^, and Gln^389^), and other closely related *Aspergillus* spp. (Figure 1B). Thus, suggesting that fungal Bags may also associate with Hsp70s.

**Figure 2:**
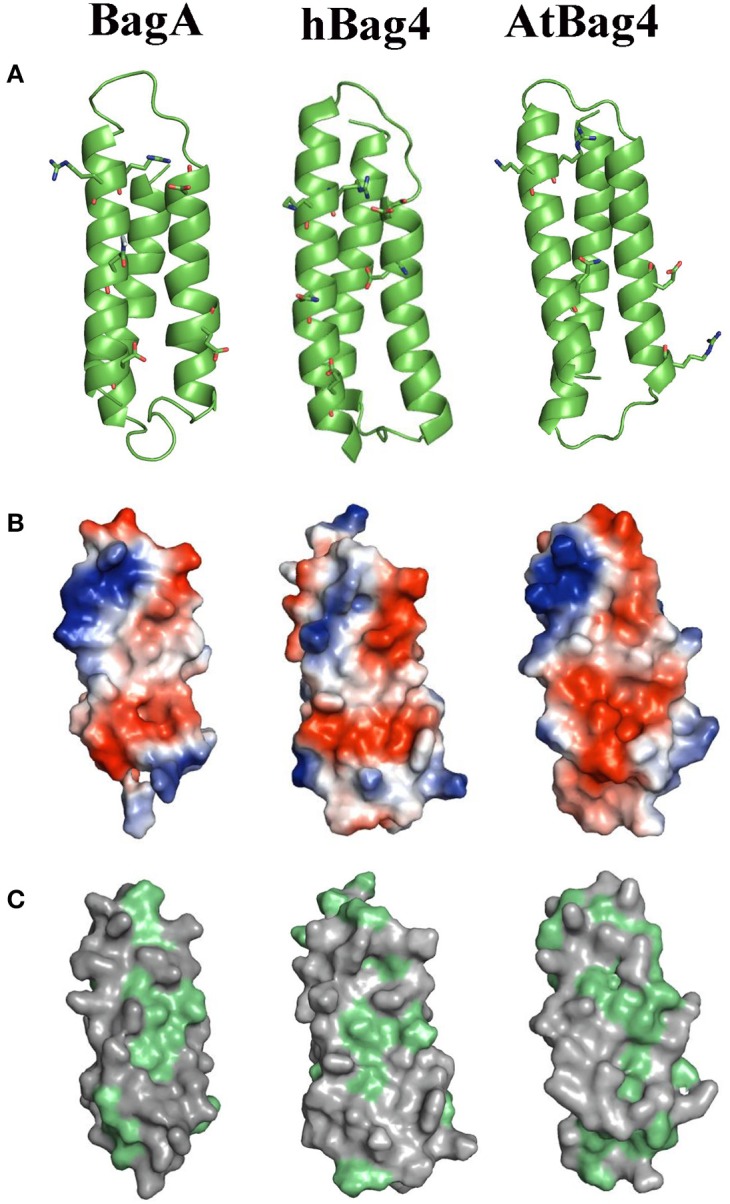
Homology modeling, electrostatic potential, and hydrophobicity of BagA. **(A)** Ribbon representation of BagA BD, homolog model is shown along with solution NMR structures of AtBag4 (Protein data bank code: 4HWH) and hBag4 (Protein data bank code: 1M7K). Green ribbons represent three anti-parallel alpha helices with second and third helices facing forward. **(B)** The hydrophilic surface is colored based on the electrostatic potential (Negative –Red to Positive- Blue). The second and third helices are orientated toward the front similar to the ribbon representation. **(C)** The surface map of hydrophobic residues is shown; hydrophobic residues are highlighted in green.

We further used protein structure-based homology modeling to generate a 3D structure of the BD of BagA using the PHYRE2 web portal (Kelley et al., 2015). The available solution NMR structure of BDs of hBag4 and AtBag4 were used as reference (Doukhanina et al., 2006). Our results indicate that the BD of BagA contains three antiparallel alpha helices similar to hBag4 and AtBag4 (Figure 2A). As previously mentioned, one key feature of BDs is their ability to form complexes with the ATPase domain of Hsp70s. We thus compared the Hsp70 binding surfaces (α2 and α3 helices) of all three BDs since they contain the key amino acid residues required for Hsp70 binding. The overall charge distribution of amino acid residues in the BagA BD is very similar to hBag4 and AtBag4 as revealed by the surface potential maps of the three BDs (Figure 2B). Specifically, the negative charge is contributed by acidic residues Glu^357^ (in the α2 helix), Asp^368^ (in the connecting loop between α2 and α3 helices), and Asp^396^ (in the α3 helix). The positive charge is contributed mostly by Arg^378^, Arg^381^, and Arg^382^ in the α3 helix of BagA BD. The hydrophobicity of the central region of the Hsp70 binding surface in hBag4 and BagA was also conserved, with amino acid residues Ile^359^, Leu^360^, Ile^365^, and Ile^385^ contributing to the hydrophobicity of BagA (Figure 2C). The α1 helix of BDs typically provides substrate specificity and overall stability to the protein. The conservation of amino acid residues in α1 helix was rather low compared to α2 and 3, which may suggest fungal specific targets for BagA. Overall, we show here that the BD of BagA shares striking similarities in 3D structures, domain organization, amino acid properties, and Hsp70 binding surfaces to animal and plant Bags. Thus, we propose that BagA is a structural and potentially a functional homolog of known Bag proteins.

The ability of BagA to bind *A. nidulans* Hsp70s was tested in yeast. BagA was cloned in the bait vector pGBKT7 (clontech's Matchmaker® Gold Yeast Two-Hybrid System) and assayed against the four annotated *A. nidulans* Hsp70s; Hsp70 (AN5129), BipA (AN2062), SgdE (AN6010), and HscA (AN12473) cloned into the prey vector pGADT7. AtBag4 and AtHsc70 served as positive control (Doukhanina et al., 2006). Despite the conservation of key Hsp70 binding residues, interaction between BagA and *A. nidulans* Hsp70s was not detected in yeast two hybrid (Figure S2). However, we cannot rule out the possibility that this association occurs under specific cellular conditions in *A. nidulans*, or that other factors may be required *in vivo*. In accordance with the yeast-two-hybrid results, mutation of a key residue (D368S) required for Hsp70 binding in animals and plants (Doukhanina et al., 2006) did not alter the function of BagA since the mutated Δ*bagA* (D368S) was able to restore the Δ*bagA* phenotype (Figure S3). Additionally, we performed *in vivo* pull-down assays using BagA::Flag (strain RSJA 18.1) as bait (Figure S4). This assay did not reveal any Hsp70s as potential BagA binding partners under the conditions tested, however, spectral-based quantification revealed several significant (greater than 80.0% probability within 1% False Discovery Rate) BagA interacting proteins that will require further characterization (Figure S4). Thus, BagA likely performs fungal-specific functions that do not rely on Hsp70 binding and suggest that regulation of Hsp70 activity may not be a universal attribute of Bag proteins.

### *bagA* expression is induced in response to apoptotic stimuli and during fungal development

**Figure 3:**
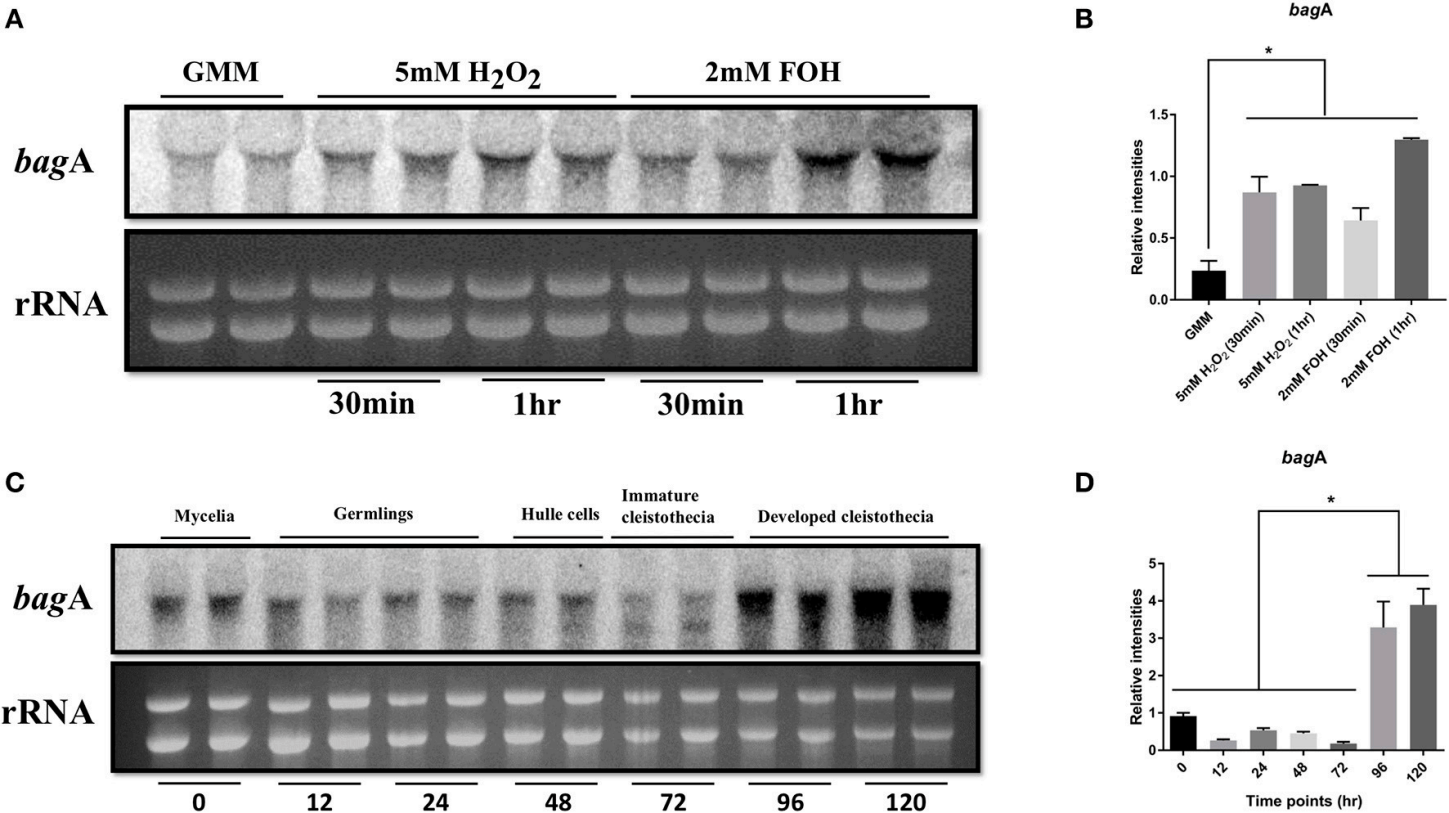
Expression analysis of *bagA*. Northern blot analysis of wild type (RJMP103.5) in response to apoptotic stress (2 mM Farnesol) and oxidative stress (2 mM H_2_O_2_) is shown. Ribosomal RNA (rRNA) was used as a loading control **(A)**. Cultures were harvested either 30 min or 1 h after adding the stressors and blots were probed with *bagA*, the bands were quantified in ImageJ and plotted for significance in Graphpad prism **(B)**. Strains were grown under sexual development inducing conditions and harvested at specified time intervals. Ribosomal RNA (rRNA) was used as a loading control. Duplicates of each time point are shown **(C)**, the bands were quantified in ImageJ and plotted for significance in Graphpad prism **(D)**. Stars (^*^) indicate statistical significance (*p* < 0.05).

The first Bag protein (hBag1) was identified in an interactor screen using the anti-apoptotic protein Bcl-2 as bait and was shown to enhance the anti-apoptotic activity of Bcl-2 (Takayama et al., 1999). Accordingly, *bagA* gene expression was investigated in the *A. nidulans* wild type strain RJMP103.5 in response to known cell death elicitors such as farnesol and oxidative stress. Farnesol induces apoptotic-like cell death in fungi and triggers apoptotic hallmarks, including DNA laddering, nuclear condensation and TUNEL positive nuclei (Semighini et al., 2006). Northern blot analysis showed that *bagA* expression was specifically and strongly induced within 1 h of farnesol exposure (Figure 3A). Transcript levels were also evaluated in response to oxidative stress imposed by hydrogen peroxide (H_2_O_2_). Reactive oxygen species (ROS) are important cell death intermediaries, and their accumulation is required for farnesol-induced apoptosis in *A. nidulans* (Semighini et al., 2006). The expression of *bagA* was again induced following 5 mM treatment with H_2_O_2_ (Figures 3A,B). This suggests that BagA may play a role in response to ROS inducing stresses and is in line with other studies proposing a protective effect for Bag proteins against oxidative damage (Doukhanina et al., 2006; Wang et al., 2014). We tested whether the deletion of *bagA* resulted in enhanced sensitivity to either farnesol or H_2_O_2_. Unexpectedly, Δ*bagA* strains were not significantly impacted by these stresses compared to wild type (data not shown). The loss of BagA may be compensated by other stress coping mechanisms in *A. nidulans*, thus explaining this result.

In addition to stress responses, *bagA* transcript accumulation was monitored throughout developmental stages ranging from vegetative growth to the sexual life stages. Transcripts were detected at various stages of development, however, *bagA* expression peaked during the sexual phase, particularly at the later stages of sexual fruiting body (cleistothecia) formation (Figures 3C,D). Bag proteins play an integral role in developmental processes in other organisms (Doukhanina et al., 2006; Rosati et al., 2007). The strong induction of *bagA* during sexual differentiation is intriguing and suggests that it may play a role in *A. nidulans* development.

### *bagA* modulates the balance between sexual and asexual development in *A. nidulans*

**Figure 4:**
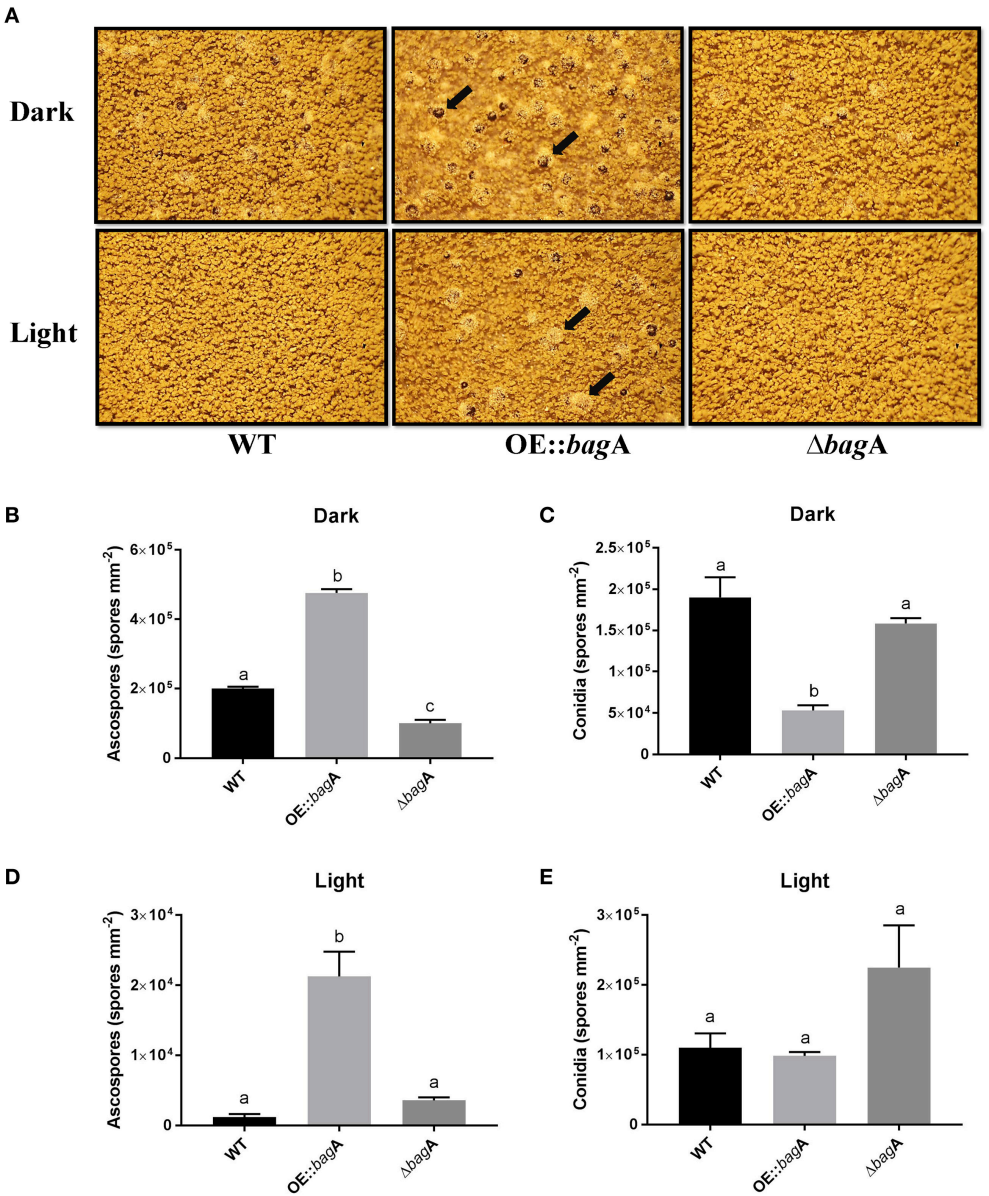
Growth phenotypes of wild type, OE::*bagA*, and Δ*bagA* strains. **(A)** Strains were grown on Glucose minimal media for 7 days at 37°C in continuous dark or light conditions. Pictures were taken on dissecting scope on 7th day. Black arrows point toward sexual fruiting bodies (cleistothecia) which contains the sexual spores (ascospores). Equal number of cores were taken and counted using hemocytometer. Means ± standard errors are indicated for three replicates of each strain. Levels not connected by same letter are significantly different (*P* < 0.05) according to the 1 way ANOVA test performed using GraphPad® prism software. Quantification of ascospores during Dark **(B)**, conidia during dark **(C)**, ascospores during light **(D)**, and conidia during light **(E)**.

In light of the expression data, a reverse genetics approach was used to further investigate the role of *bagA* during sexual development. *bagA* deletion (Δ*bagA*) and overexpression (OE::*bagA*) strains were generated in the *A. nidulans* wild type background RJMP103.5. The integrity of these strains was confirmed using southern and northern blotting (Figures S5A,B). The ability of the generated strains to produce sexual fruiting bodies and ascospores was monitored in solid glucose minimal media (GMM) kept in dark at 37°C (conducive conditions for sexual development) (Tsitsigiannis et al., 2004). On solid GMM, the OE::*bagA* strain produced significantly more cleistothecia and ascospores compared to wild type (Figures 4A,B, Figure S6). In contrast, the deletion of *bagA* resulted in markedly lower fruiting structures and ascospore numbers (Figures 4A,B, Figure S6). Wild type levels of sexual sporulation were restored following complementation of the Δ*bagA* strain (Figure S3). Interestingly, conidiation was also significantly impacted in OE::*bagA*, and was inversely correlated with ascospore production (Figure 4C). Furthermore, the capacity of the OE::*bagA* strain to produce cleistothecia and ascospores is maintained under light, a condition that typically suppresses sexual development (Figures 4D,E).Taken together, these results show that BagA is involved in mediating the balance between sexual and asexual development, and is a positive regulator of sexual fruiting body formation.

**Figure 5:**
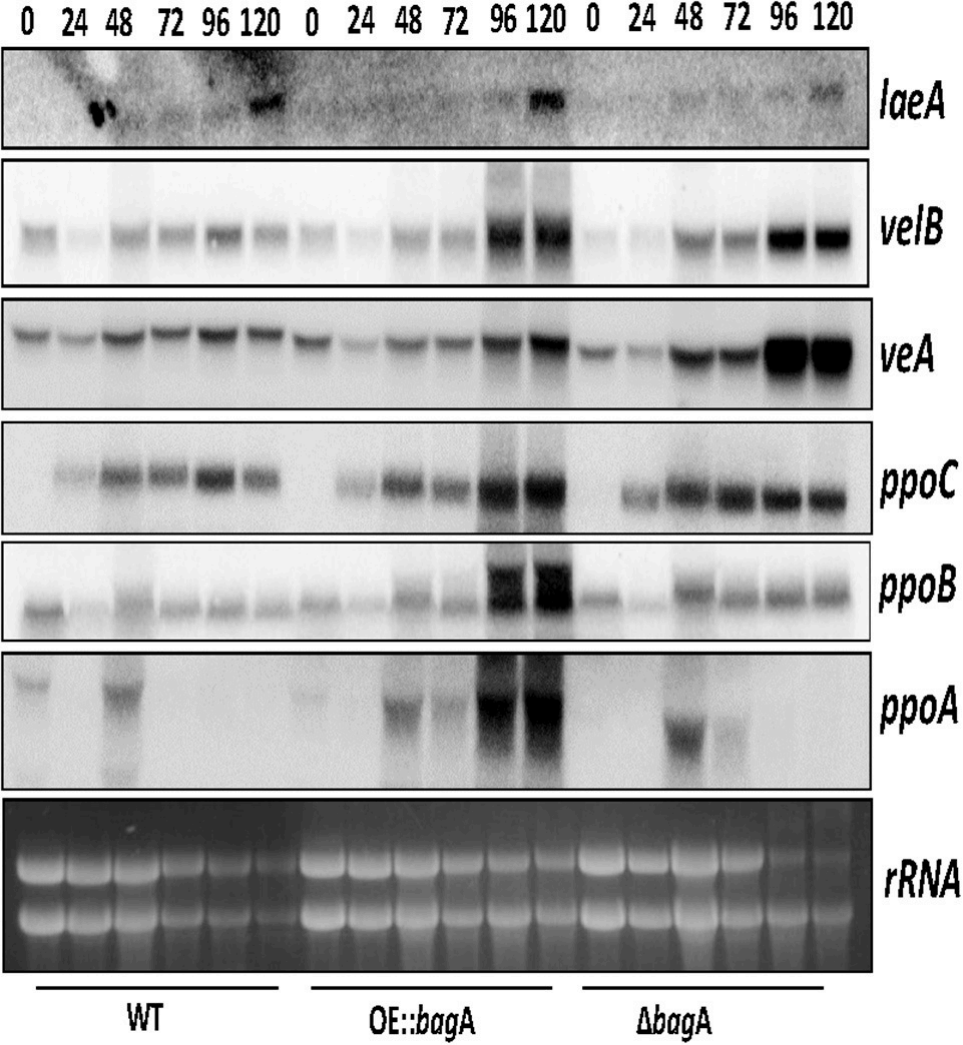
Gene expression analysis of *ppoA*BC and velvet complex members. Northern blot analysis of strains; WT, OE::*bagA*, and Δ*bagA* grown under sexual development inducing conditions and harvested at specified time intervals indicated in hours post-induction of sexual development. Ethidium bromide-stained rRNA are included as loading control.

Although, the pathways dictating the sexual/asexual developmental balance in *A. nidulans* are inherently complex, one family of proteins called fungal dioxygenases (Ppo proteins) are well known for their modulating effects on development. We were intrigued by the phenotypic parallel between Δ*bagA* and that of Δ*ppoA*, a positive regulator of sexual development in the Ppo family of proteins. Akin to *bagA*, the deletion of *ppoA* showed a significant reduction in sexual sporulation, while its overexpression had the opposite effect (Tsitsigiannis et al., 2004). We determined *ppoA* transcript levels in the Δ*bagA* and OE::*bagA* strains, and found a much stronger induction of *ppoA* expression in the OE::*bagA* background, particularly at the later stages of cleistothecial development (Figure 5; Figure S7). We included *ppoC* and *ppoB* in this transcript analysis, two other dioxygenases that integrate sexual and asexual development in *A. nidulans*, and are antagonistic to *ppoA* (Tsitsigiannis et al., 2005). Normalized *ppoC*/*ppoB* levels remained largely unchanged between Δ*bagA* and OE::*bagA* (Figure 5; Figure S7), thus the higher transcript ratio of *ppoA* to *ppoC*/*ppoB* in OE::*bagA* could be responsible for the strong and constitutive push toward sexual development. We thus asked whether the overexpression of *ppoA* can compensate for the defect imposed by the *bagA* deletion. However, ascospore levels produced by the OE::*ppoA*/Δ*bagA* strain were statistically indistinguishable from Δ*bagA*, and remained markedly lower than the OE::*bagA* strain (Figure S8). These results suggest that BagA regulation of sexual development in *A. nidulans* involves factors other than PpoA. The expression of other know developmental regulators in *A. nidulans* were also tested, including BrlA (a positive regulator of asexual conidiation) and NsdD (a positive regulator of sexual development) (Lee et al., 2016). However, no distinct patterns of expression that may explain the observed phenotypes were detected (Figure S9).

We considered the role of velvet complex members (VeA, VelB, and LaeA) in the sexual phenotype imposed by the overexpression of *bagA*. VeA in particular is known to activate sexual development in *A. nidulans* (Kim et al., 2002). We thus examined the expression levels of *ve*A, *vel*B, and l*ae*A in our Δ*bagA* and OE::*bagA* strains. Surprisingly, despite the sexual development defect in Δ*bagA, ve*A expression was induced at higher levels in this strain at 96 and 120 h post-induction of sexual development, while *vel*B and *lae*A transcripts remained largely unchanged across strains (Figure 5; Figure S7). We speculate that *ve*A may be induced at higher levels as a compensating mechanism for the aberrant sexual development caused by the *bagA* deletion.

### Deletion of *bagA* leads to global increase in secondary metabolite production

**Figure 6:**
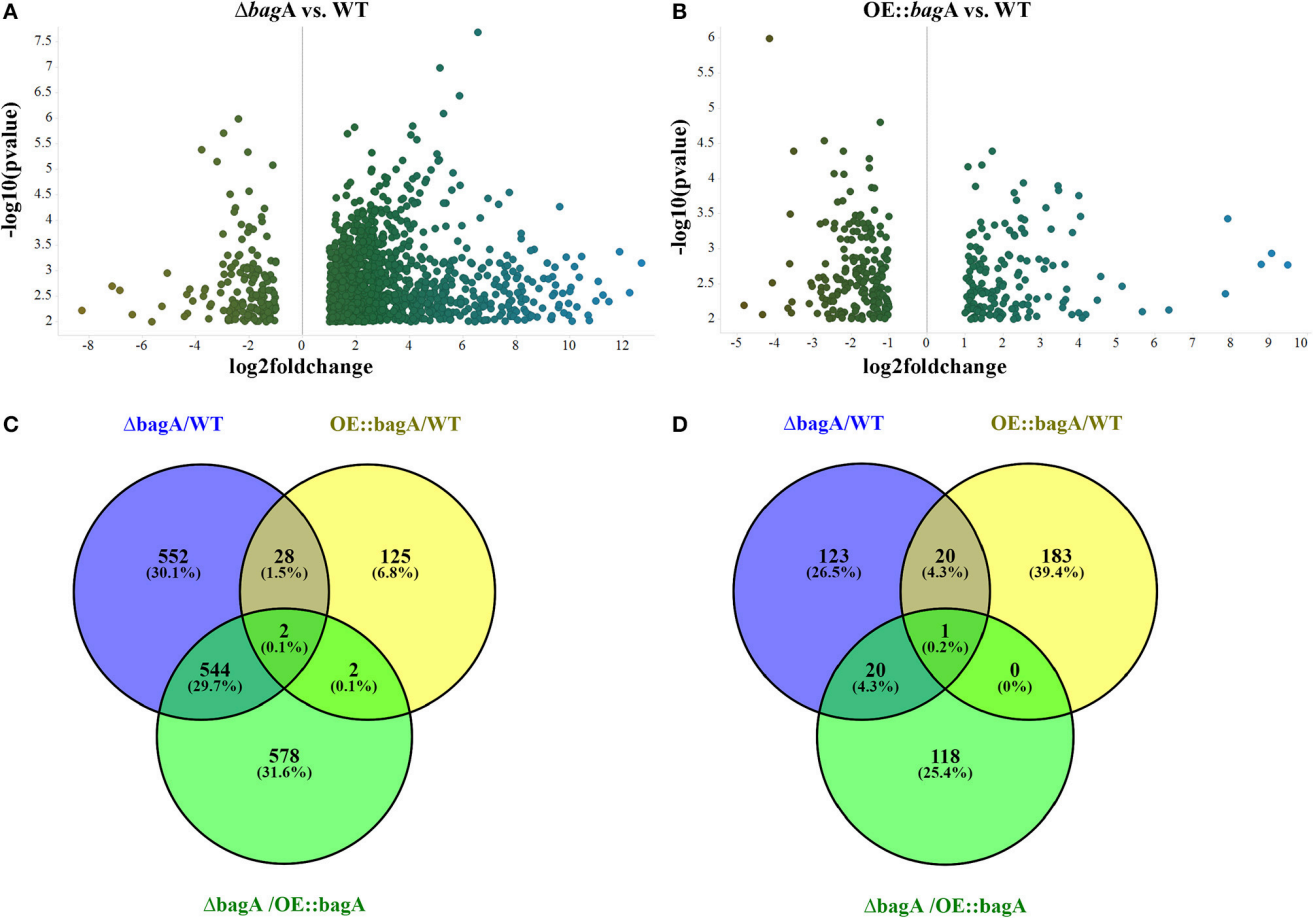
Differentially regulated metabolites in OE::*bagA* and Δ*bagA* compared to wild type in the vegetative phase. **(A,B)** Volcano plots of differentially regulated metabolites are shown. Pairwise comparisons were performed comparing OE::*bagA* and Δ*bagA* to wild type. **(C,D)** represent total upregulated and downregulated metabolites produced during vegetative growth phase, respectively. Analysis was performed using minimum threshold of fold change >2 and *p* < 0.01.

**Figure 7:**
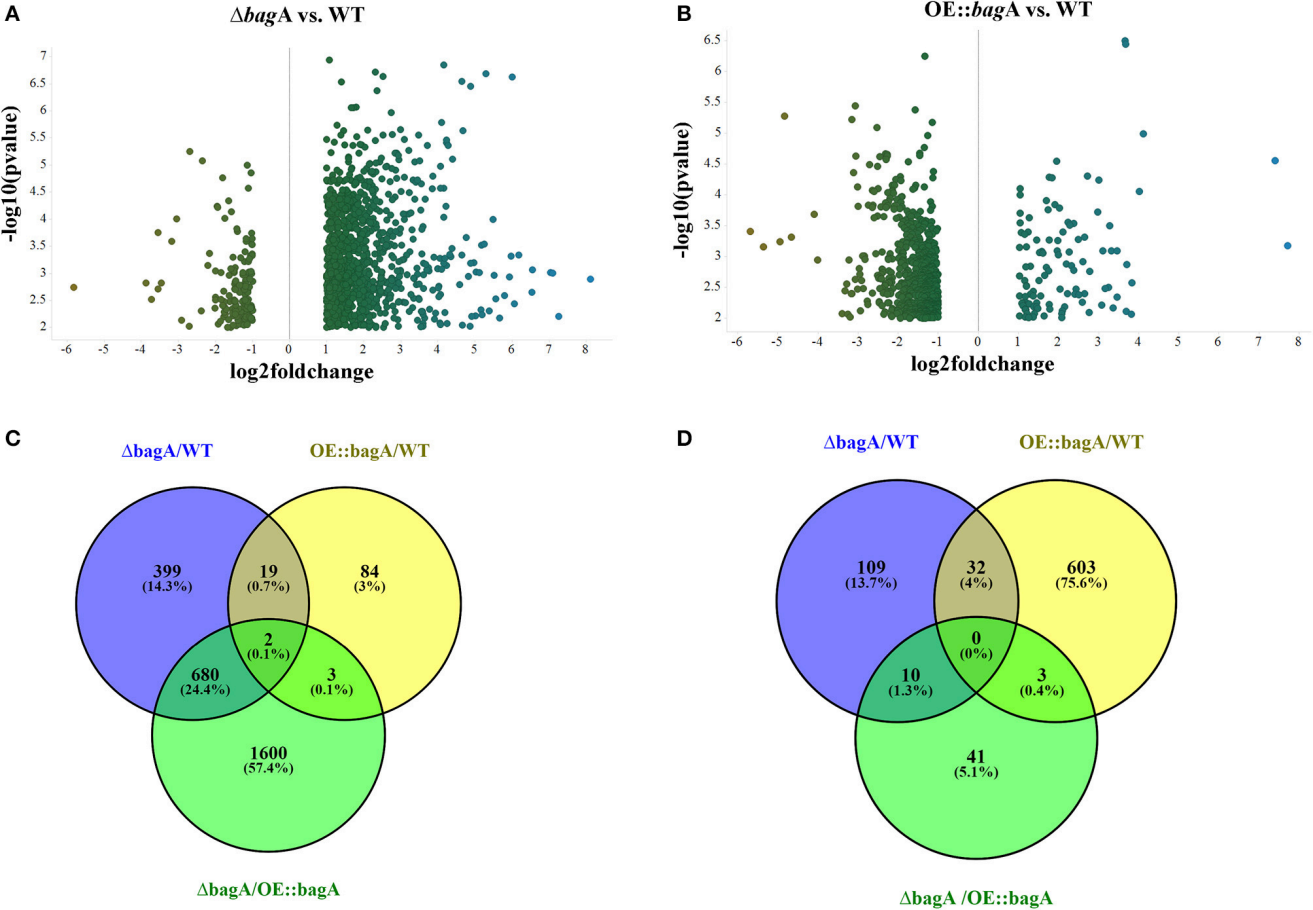
Differentially regulated metabolites in OE::*bagA* and Δ*bagA* compared to wild type in sexual development phase. **(A,B)** Volcano plots of differentially regulated metabolites are shown. Pairwise comparisons were performed comparing OE::*bagA* and Δ*bagA* to wild type. **(C,D)** represent total upregulated and downregulated metabolites produced during sexual growth phase respectively. Analysis was performed using minimum threshold of fold change >2, and *p* < 0.01.

During the phenotypic analysis of the Δ*bagA* and OE::*bagA* strains, we observed distinct pigmentation changes in Δ*bagA* liquid cultures (Figure S10A). We reasoned that such a change could be due to an altered secondary metabolite (SM) profile. Interestingly, several studies have reported an intimate link between fungal sexual/asexual development and secondary metabolism (Bayram et al., 2008, 2016; Nahlik et al., 2010). Accordingly, we performed unbiased metabolomics analysis to compare the global SM profiles of OE::*bagA*, Δ*bagA*, and wild type strains under various growth conditions. SMs were extracted using appropriate solvents from liquid culture supernatant (vegetative phase) or fungal tissues scraped off from solid media (sexual phase). LC-MS analysis generated over 1,000 features, peak detection and relative quantification were achieved using XCMS online (Tautenhahn et al., 2012). Scatter plots were generated in Spotfire graphical program (TIBCO®) for statistically significant features (*p* < 0.01) with absolute fold changes greater than two. Compared to wild type, Δ*bagA* was enriched in more than 500 metabolites compared to wild type in both vegetative (Figures 6A–D) and sexual growth conditions (Figures 7A,C). The reverse trend was observed in the OE::*bagA* strain, with more downregulated metabolites compared to wild type, particularly in the sexual phase (Figures 7B,D). Although there is still limited information on the ID of these metabolites, we used an in-house database partly generated using Reaxys (repository of chemicals from known sources) to putatively annotate known *A. nidulans* SMs based on exact mass (Barkal et al., 2016). Metabolites were identified using specific criteria (fold change ≥ 2, *p* ≤ 0.01, and ppm error ≤ 5) and linked to their respective SM cluster (Table 2). Of note, the Δ*bagA* strain accumulated significantly higher levels of dehydroaustinol, a meroterpenoid linked to sporulation in *A. nidulans* (Rodríguez-Urra et al., 2012). It is important to mention that some of the metabolites that were putatively annotated as secondary metabolites produced by *A. nidulans* were cryptic SMs under standard growth conditions (Bok et al., 2009). In addition to the known metabolites, there are still a large number of features that remain unidentified including novel metabolites only associated with the Δ*bagA* strain (Table 3).

**Table 2:** Table of predicted metabolites differentially expressed in Δ*bagA* compared to wild type.

**Condition**	**Cluster backbone gene ID**	**End product of cluster**	Putative metabolite^*^	**m/z**	**Log2 Fold**
Sexual	ANID_00150	Monodictyphenone	2-OHEM or “-OHEM (citreorosein)”	285.0406	4.38333
			“Astochrysone carboxylic acid”	405.0837	2.469048
			“Variecoxanthone A”	339.1239	4.88103
	ANID_08383	Austinol	“Dehydroaustinol”	501.1774	4.884
			“Isoaustinone or 5”R-isoaustinone”	471.2031	3.451657
			“Neoaustinone or austinolide or 11beta-hydroxyisoaustinone”	441.1923	2.042041
			“Preaustinoid A3, A4, or A5”	491.1851	2.141732
			“Protoaustinoid A”	429.2649	2.228574
	ANID_02545	Emericellamide A	“Emericellamide C/D”	630.365	2.847332
	ANID_07071	Alternariol	“Alternariol”	515.099	2.29207
Vegetative phase	ANID_00150	Monodictyphenone	“Monodictyphenone”	333.0616	5.316391
			“2,w-hydroxyemodin”	347.0409	4.431758
			“Variecoxanthone A”	385.1293	4.264474
	ANID_08383	Austinol	“Preaustinoid A3, A4, or A5”	501.2130	10.50882
			“Austinoneol”	413.1969	2.610656
			“Preaustinoid A3, A4, or A5”	455.2075	2.604056
			“Isoaustinone or 5”R-isoaustinone”	425.1969	1.347772
			“Neoaustinone or austinolide or 11beta-hydroxyisoaustinone”	441.1919	1.332291
			“Isoaustinone or 5”“R-isoaustinone”	425.1969	1.136842
	ANID_08513	Terrequinone A	“Terrequinone A”	489.2183	2.8695
	ANID_02545	Emericellamide A	“Emericellamide A”	608.4028	1.476079
	ANID_07909	F-9775	“3-methylorsellinic acid”	363.1085	1.564688
			“3,5-dimethylorsellinic acid or 2,4-dihydroxy-3,5,6-trimethylbenzoic acid”	195.0663	2.167813
			“6-ethyl-2,4-dihydroxy-3,5-dimethylbenzaldehyde”	387.1813	1.526432
			“2,4-dihydroxy-3,5,6-trimethylbenzaldehyde”	225.0768	1.473577

* *The known secondary metabolites from A. nidulans were putatively identified with allowed mass error of 5.0 ppm*.

**Table 3:** A list of unique features only identified in the Δ*bagA* strain.

**Feature id**	**m/z**	Mean peak area^*^	***p*-value**
534	155.1429	329460.6065	0.001278
428	186.1207	140902.6194	0.000889
1644	328.0828	317369.089	0.009052
712	360.0516	230886.6571	0.002216
1028	369.2645	1426108.159	0.004039
1265	467.9998	121743.1224	0.00581
248	563.3289	185212.3541	0.000394
1140	617.2197	103346.457	0.004848
353	623.2865	3302707.428	0.000691
842	624.2894	1209003.84	0.002948

* *Area of the peak corresponding to a mass (mean of three replicates)*.

The velvet complex composed of VeA, VelB, and LaeA is known to regulate secondary metabolism in *A. nidulans* (Bayram et al., 2008). We assessed the transcript level of all three members in wild type, OE::*bagA*, and Δ*bagA* strains. The expression levels of *ve*A, *vel*B, and *lae*A were unaffected in these strains (Figure S10B). Thus, the deletion of *bagA* creates a cellular environment that is more conducive to the accumulation of secondary metabolites through a yet to be determined mechanism.

## Discussion

The evolutionary conserved Bag family of proteins regulates a remarkably array of cellular functions. To date, Bag studies have largely focused on mammalian and plant systems. Herein, we report the discovery and characterization of a Bag protein in the model filamentous fungus *Aspergillus nidulans*, designated BagA, using a combination of bioinformatic tools, expression studies, reverse genetic approaches, and mass spectrometry. Several lines of evidence are consistent with the following conclusions: (1) *A. nidulans* encodes a single Bag family member, BagA. (2) BagA is a bona fide Bag protein that shares striking similarities in 3D structures, domain organization, and amino acid properties with its animal and plant counterparts. (3) The expression of *bagA* is induced in response to environmental insults. (4) BagA modulates sexual development in *A. nidulans*. (5) BagA regulates, either directly or indirectly, secondary metabolite production. Overall, our results indicate that some aspects of Bag regulation are conserved in filamentous fungi and implicate Bag in both developmental processes and secondary metabolism. To our knowledge, this study provides a first insight into Bag function in filamentous fungi.

Bag proteins are distinguished by a common conserved region known as the Bag domain (BD). Using BD sequences however, common BLAST approaches using BD sequences however, fail to reliably identify Bag homologs in other taxa due to sequence divergence among these functionally related proteins (Doukhanina et al., 2006). Here we use more sensitive protein domain search tools such as HMM-based Pfam models to identify Bag domain containing proteins in fungi. Using this approach, we identified putative Bag homologs across various fungal phyla, including pathogenic and non-pathogenic fungi. Domain organization of most fungal Bags, including *Aspergillus nidulans* BagA, shares commonalities with the human Bag1 (hBag1) and *Arabidopsis* Bag1 through 4 (AtBag1-4) with the conserved C-terminal BD and an N-terminal Ubiquitin-like (UBL) domain. Using homology modeling of BD, charge characteristics, and hydrophobicity features, we show that BagA BD shares striking similarities with plant and animal Bag proteins, indicating that BagA is a bona fide structural homolog of its animal and plant counterparts. A distinguishing feature of the BD is its ability to bind the ATPase domain of Hsp70/Hsc70 molecular chaperones, and in some cases regulate their function (Takayama et al., 1999; Doukhanina et al., 2006; Kabbage and Dickman, 2008; Kabbage et al., 2017). Our analysis of the BD showed that the key residues required for Hsp70 binding are largely conserved in BagA. Thus, suggesting that BagA may also cooperate with *A. nidulans* Hsp70s. Surprisingly, our experimental evidence shows that BagA does not interact with *A. nidulans* Hsp70s in yeast, and mutations within the presumed Hsp70 binding surface did not alter BagA function. We propose that Hsp70 binding and regulation may not be a universal feature of Bag proteins. This is supported by results in *Arabidopsis* where AtBag6, a protein required for autophagy and fungal resistance (Li L. et al., 2016), did not interact with AtHsc70 (Kang et al., 2006). Similarly, the human Bag6 does not act as other BD containing proteins to cooperate with Hsp70 (Mock et al., 2015). However, we cannot rule out that this association occurs under specific conditions in *A. nidulans*.

Studies in *A. nidulans* showed that programmed cell death (PCD) with apoptotic features can be induced via application of chemicals such as farnesol and hydrogen peroxide. Farnesol is a quorum sensing molecule produced by *Candida albicans* that was shown to trigger PCD in filamentous fungi, including *A. nidulans*. Farnesol induced cell death requires the generation of reactive oxygen species (Semighini et al., 2006). Here we report that exposure to either farnesol and hydrogen peroxide strongly induces *bagA* expression. While other Bags are established anti-apoptotic/PCD proteins (Kabbage and Dickman, 2008; Kabbage et al., 2017), surprisingly, no significant effects were observed in response to these PCD inducing stimuli in our *bagA* deletion strain compared to wild type. We attribute this unexpected result to the complexity of cell death and stress responses in fungi and other organisms, and stipulate that other cytoprotective compensatory mechanisms are being activated in response to these insults.

An important function of Bag proteins is their ability to influence developmental processes (Briknarová et al., 2001; Doukhanina et al., 2006). Indeed, the effect of the human Bag1 on cell growth and its association with hormone and retinoic acid receptors is well documented (Matsuzawa et al., 1998). More recently, the interplay of human Bag1 and Bag3 was shown to affect aging processes through the regulation of protein degradation (Gamerdinger et al., 2009). In mammalian development, the deletion of *bag*6 was linked to severe developmental defects in the lung, kidney, and brain (Desmots et al., 2005). The influence of Bags on development in maintained in plants, *Arabidopsis bag*4 and 6 knock-outs exhibited altered flowering phenotypes, shorter vegetative and reproductive phases, and accelerated senescence (Doukhanina et al., 2006). A recent study has also linked *Arabidopsis* Bag5 to senescence (Li L. et al., 2016). This developmental aspect of Bag regulation appears to be conserved in *A. nidulans*. Specifically, BagA regulates the balance between asexual and sexual reproduction. The expression of *bagA* was drastically induced during sexual development, and its overexpression (OE::*bagA*) promoted sexual fruiting body formation (cleistothecia) and sexual spore (ascospores) production, this phenotype was accompanied by a reduction in asexual spore (conidia) formation. Furthermore, the OE::*bagA* strain produced significantly more ascospores under light, a condition that is typically non-permissive to sexual development, indicating a constitutive push toward sexual body formation. Conversely, the *bagA* null mutant was impaired in sexual structure formation but displayed increased conidiation. An important question is how does BagA shift the balance toward sexual development? Three fatty acid dioxygenases (PpoA, PpoB, and PpoC) are known to modulate sexual and asexual balance in *A. nidulans* (Tsitsigiannis et al., 2004, 2005). Specifically, *ppoA* overexpression was shown to positively affect ascosporogenesis and negatively regulate conidiation (Tsitsigiannis et al., 2004). Phenotypes that we observed in our OE::*bagA* strain. Interestingly, *ppoA* expression was strongly induced in OE::*bagA* thus potentially explaining the sexual phenotype in this strain. However, the overexpression of *ppoA* in the Δ*bagA* background did not restore the sexual development defect in Δ*bagA*, suggesting that other factors are implicated.

Global metabolomics profiles revealed a clear increase in SM output associated with the deletion of *bagA*. Indeed, the Δ*bagA* strain was enriched in over 500 metabolites compared to wild type. This was an interesting result considering the previously established link between SM and asexual/sexual development (Bayram et al., 2016), and suggest that BagA, directly or indirectly, modulates secondary metabolism in *A. nidulans*. Bag proteins are known to interact with transcription factors, the proteasome, and cell cycle regulators, and are proposed to function as molecular rheostats orchestrating cell state depending on whether the environment is stressful or favorable (Briknarová et al., 2001; Kabbage and Dickman, 2008; Kabbage et al., 2017). The deletion of *bagA* may thus alter cellular homeostasis causing the production of atypical or stress metabolites. The UBL domain in BagA and other Bag proteins suggest involvement with proteosome-mediated protein regulation. Indeed, the human Bag1 was reported to cooperate with an E3 ligase, CHIP, to target proteins for degradation (Ballinger et al., 1999; Demand et al., 2001). Conversely, the human Bag2 was shown to inhibit CHIP ubiquitin ligase activity (Dai et al., 2005). This dual function of Bags is consistent with their presumed role as molecular switches. In fungi, the conserved COP9 signalosome (CSN) multiprotein complex regulates proteasome-mediated protein turnover (Busch et al., 2003). Recently, *csnE* mutants displayed altered SM output as a result of improper functioning of the proteasome degradation pathway (Gerke et al., 2012). Though a direct link between BagA and the proteasome is yet to be established, it is reasonable to speculate that such misregulation may also occur in our *bagA* strains resulting in abnormal SM production. Regardless of how BagA modulates secondary metabolism in *A. nidulans*, we propose that perturbing cellular homeostasis can be used as a strategy to unlock the SM potential of filamentous fungi. As shown here with the deletion of *bagA*, such strategy can lead to increased production of valuable metabolites, but importantly, activate silent clusters and facilitate the discovery of novel metabolites most assuredly to possess.

## Author contributions

SJ, NK, and MK developed the study and designed the experiments. SJ, PW, and ET performed the research. SJ, PW, BW, NK, and MK analyzed and interpreted the data. SJ and MK wrote the manuscript. MK is the corresponding author on this manuscript. All authors reviewed the manuscript.

### Conflict of interest statement

The authors declare that the research was conducted in the absence of any commercial or financial relationships that could be construed as a potential conflict of interest.

## Supplementary material

Figure S1 Phylogenetictree by maximum likelihood method of Bag proteins. BD of Bag proteins from representative species of different taxonomic groups were aligned using MEGA6. Multiple sequence alignment of BDs was used to construct neighbor joining tree (bootstraps = 1,000) in Mega6. Major phyla and kingdoms are color coded.

Figure S2 Yeast-two-hybrid assay. Yeast strain AH109 was transformed with the indicated bait and prey plasmids. BagA was used as bait and assayed against the four annotated Hsp70s from *A. nidulans*; Hsp70 (AN5129), BipA (AN2062), SgdE (AN6010), HscA (AN12473). *Arabidopsis* AtBag4 and AtHsc70 were used as a positive control. BagA with empty prey vector (EV) served as negative control.

Figure S3 Complementation of the Δ*bagA* strain. Wild type, Δ*bagA*, Bag-Full-C (full length *bagA* complementation), and D368S (full length *bagA* with mutation at a critical critical residue required for Hsp70 binding) were assessed for ascospore production. Strains were grown on Glucose minimal media for 7 days at 37°C in continuous dark conditions. Equal number of cores were taken and counted using hemocytometer. Means ± standard errors are indicated for three replicates of each strain. Levels not connected by same letter are significantly different (*P* > 0.05) according to the 1 way ANOVA test performed using Graph Pad prism software. (n.s. = not significant).

Figure S4 Pull-down assay using BagA::Flag as bait. **(A)** Silver stain of the eluted protein complexes using anti-FLAG M2 magnetic beads of both WT and BagA::Flag strains are shown. Lane 1: protein ladder, Lane 2: wild type control, Lane 3 and 4: BagA::Flag strains. Arrow indicates unique band (likely ANIA_06241 shown in **B**) in the BagA::Flag compared to wild type control. **(B)** Table of identified BagA interactors. Protein identifications were accepted if they could be established at greater than 80.0% probability within 1% False Discovery Rate and contained at least 2 identified peptides.

Figure S5 Southern and northern blot analyses of the OE::*bagA* and Δ*bagA* strains. **(A)** Genomic DNA of all selected transformants and wild type was digested with AvrII and SacII. Expected hybridization band patterns for AvrII: 4.6 kb (wild type), 5.3 kb, and 2.9 kb (OE::*bagA*) strain. Expected hybridization band patterns for SacII: 8 kb, and 3.6 kb (wild type), and 12 kb (Δ*bagA*) strains. **(B)** Northern blot analysis of WT, OE::*bagA*, and Δ*bagA* grown liquid GMM for 18 h. Two transformants of each strain were loaded in duplicates. Ethidium bromide-stained rRNA are included as loading control.

Figure S6 Quantification of sexual fruiting bodies (Cleistothecia). Strains were grown on Glucose minimal media for 7 days at 37°C in continuous dark. Pictures were taken on dissecting scope and cleistothecia numbers were counted using ImageJ from the same area for each strain. Means ± standard errors are indicated for three replicates of each strain. Levels not connected by same letter are significantly different (*P* < 0.05) according to the 1 way ANOVA test performed using GraphPad® prism software.

Figure S7 Band intensities from Northern analysis of *ppoA*,B,C and velvet complex members (Figure 5) were quantified in ImageJ, normalized to the rRNA loading control, and the relative intensity was plotted in Graphpad® prism software. Numbers on the X-axis correspond to the time point post-induction of sexual development.

Figure S8 **(A)** The integrity of OE::*ppoA*; Δ*bagA* strain was confirmed by northern blotting. Integrity of OE::*bagA* and Δ*bagA* was confirmed previously (Figure S3). Ethidium bromide-stained rRNA are included as loading control. **(B)** Wild type, OE::*bagA*, Δ*bagA*, and OE::*ppoA*; Δ*bagA* were assessed for their sexual sporulation. Strains were grown on Glucose minimal media for 6 days at 37°C under continuous dark conditions. Equal number of cores were taken and ascospores counted using a hemocytometer. Means ± standard errors are indicated for three replicates of each strain. Levels not connected by same letter are significantly different (*P* < 0.05) according to the 1 way ANOVA test performed using Graph Pad prism software.

Figure S9 Gene expression analysis of *brlA* and *nsdD*. Northern blot analysis of strains; WT, OE::*bagA*, and Δ*bagA* grown under sexual development inducing conditions and harvested at specified time intervals indicated in hours post-induction of sexual development. Ethidium bromide-stained rRNA are included as loading control.

Figure S10 **(A)** Wild type, OE::*bagA*, and Δ*bagA* strains were grown in liquid GMM for 5 days at 250 rpm, and 30°C. Pictures were taken on the 5th day to indicate the pigmentation of Δ*bagA* cultures. **(B)** Expression analysis of velvet complex members under vegetative growth conditions is shown. WT, OE::*bagA*, and Δ*bagA* were loaded in triplicates and probed with gene specific primers. *bagA* was included as a control. Ethidium bromide-stained rRNA are included as loading control.

Table S1 List of strains used in this study.

Table S2 List of Primers used in this study.
