# Supplementary material for: A Bcl-2 Associated Athanogene (bagA) Modulates Sexual Development and Secondary Metabolism in the Filamentous Fungus Aspergillus nidulans
Source: Front Microbiol. 2018 Jun 15;9:1316. doi: 10.3389/fmicb.2018.01316 (PMC6013550; doi:10.3389/fmicb.2018.01316)
Supplement: Figure S1 — Phylogenetictree by maximum likelihood method of Bag proteins. BD of Bag proteins from representative species of different taxonomic groups were aligned using MEGA6. Multiple sequence alignment of BDs was used to construct neighbor joining tree (bootstraps = 1,000) in Mega6. Major phyla and kingdoms are color coded. [file Presentation_1.PDF]

Fig S1.

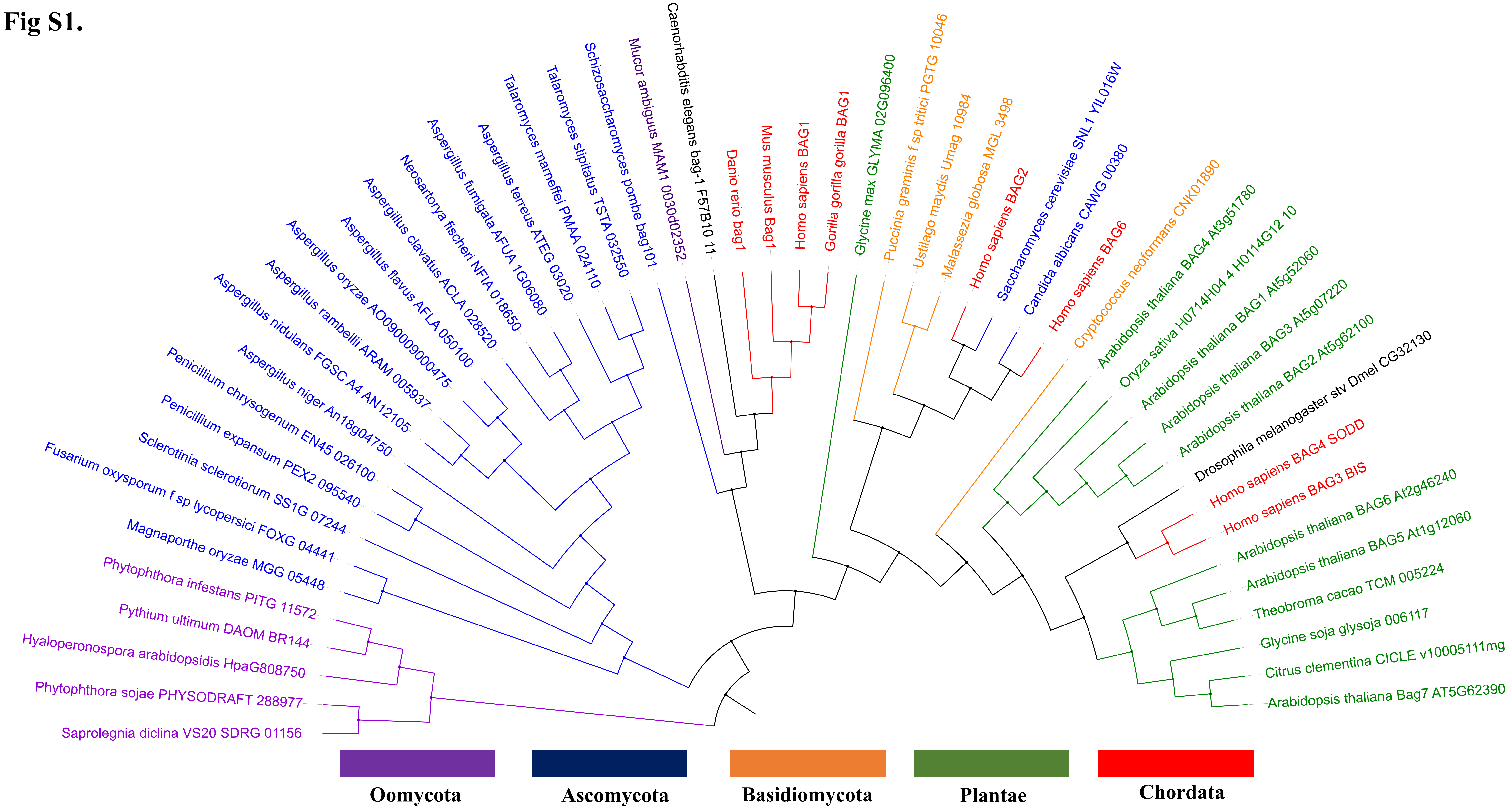

Phytophthora infestans PITG 11572
Pythium ultimum DAOM BR144
Hyaloperonospora arabidopsidis HpaG808750
Phytophthora sojae PHYSODRAFT 288977
Saprolegnia diclina VS20 SDRG 01156
Magnaporthe oryzae MGG 05448
Fusarium oxysporum f sp lycopersici FOXG 04441
Sclerotinia sclerotiorum SS1G 07244
Penicillium expansum PEX2 095540
Penicillium chrysogenum EN45 026100
Aspergillus niger An18g04750
Aspergillus nidulans FGSC A4 AN12105
Aspergillus rambellii ARAM 005937
Aspergillus oryzae AO090009000475
Aspergillus flavus AFLA 050100
Aspergillus clavatus ACLA 028520
Neosartorya fischeri NFIA 018650
Aspergillus fumigata AFUA 1G06080
Aspergillus terreus ATEG 03020
Talaromyces marneffei PMAA 024110
Talaromyces stipitatus TSTA 032550
Schizosaccharomyces pombe bag101
Mucor ambiguus MAM1 0030d02352
Caenorhabditis elegans bag-1 F57B10 11
Danio rerio bag1
Mus musculus Bag1
Homo sapiens BAG1
Gorilla gorilla gorilla BAG1
Glycine max GLYMA 02G096400
Puccinia graminis f sp tritici PGTG 10046
Ustilago maydis Umag 10984
Malassezia globosa MGL 3498
Homo sapiens BAG2
Saccharomyces cerevisiae SNL1 YIL016W
Candida albicans CAWG 00380
Homo sapiens BAG6
Cryptococcus neoformans CNK01890
Arabidopsis thaliana BAG4 At3g51780
Oryza sativa H0714H04 4 H0114G12 10
Arabidopsis thaliana BAG1 At5g52060
Arabidopsis thaliana BAG3 At5g07220
Arabidopsis thaliana BAG2 At5g62100
Drosophila melanogaster stv Dmel CG32130
Homo sapiens BAG4 SODD
Homo sapiens BAG3 BIS
Arabidopsis thaliana BAG6 At2g46240
Arabidopsis thaliana BAG5 At1g12060
Theobroma cacao TCM 005224
Glycine soja glysoja 006117
Citrus clementina CICLE v10005111mg
Arabidopsis thaliana Bag7 AT5G62390
Oomycota
Ascomycota
Basidiomycota
Plantae
Chordata

Fig S2.

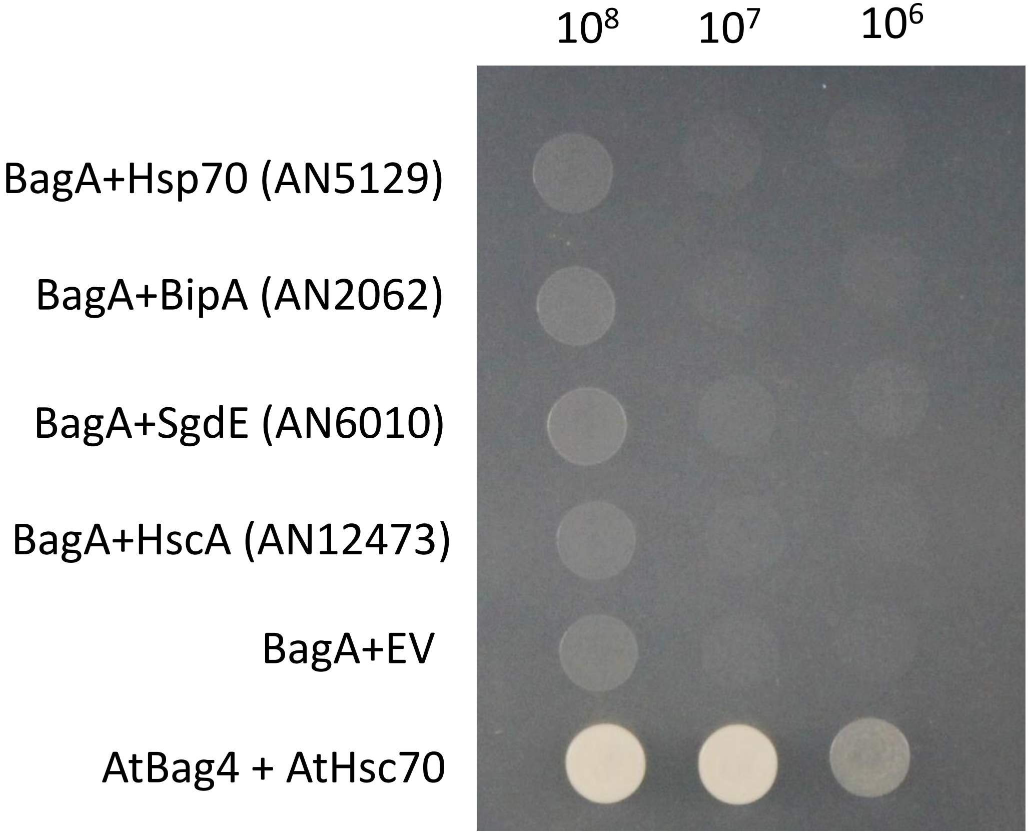
10^8
10^7
10^6
BagA+Hsp70 (AN5129)
BagA+BipA (AN2062)
BagA+SgdE (AN6010)
BagA+HscA (AN12473)
BagA+EV
AtBag4 + AtHsc70

Fig S3.

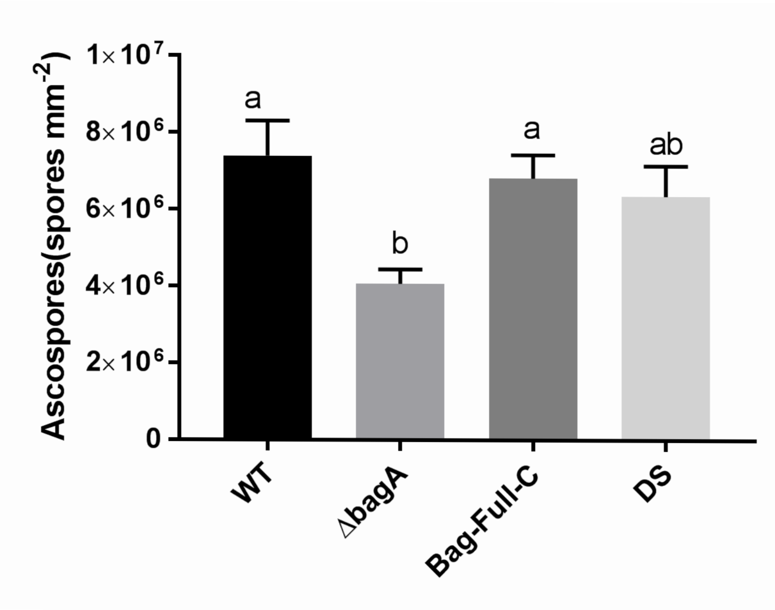

Ascospores(spores mm-2)
1×10^7
8×10^6
6×10^6
4×10^6
2×10^6
0
a
b
a
ab
WT
ΔbagA
Bag-Full-C
DS

Fig S4.

A

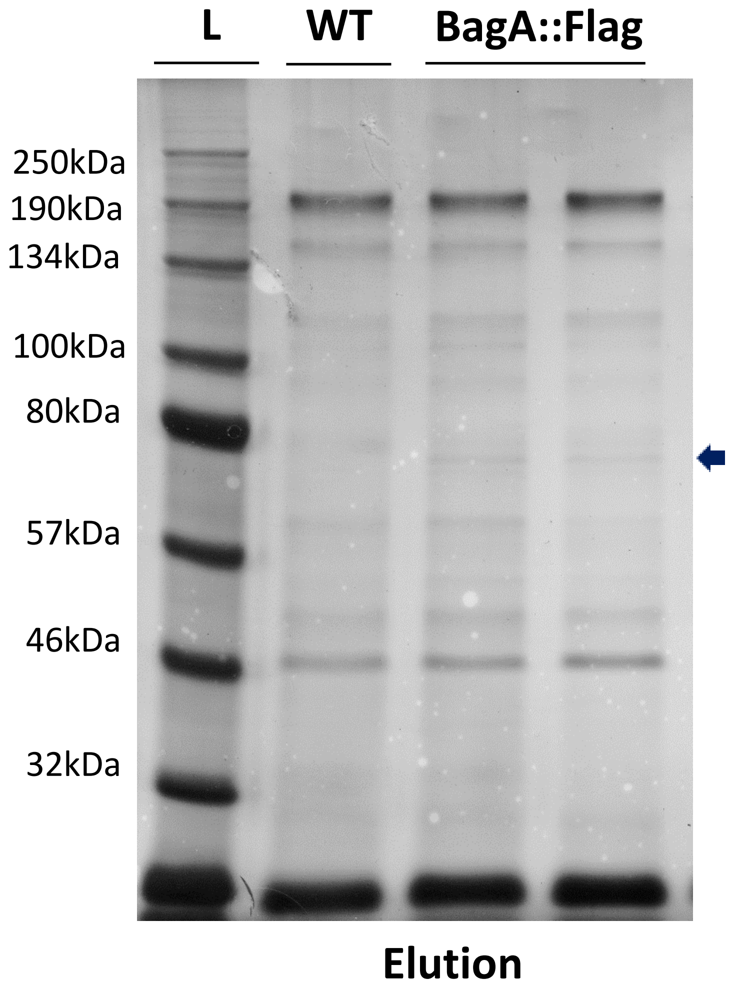

B

#	Identified Proteins	Mass	Spectral counts	
			Flag	WT
1	Uncharacterized protein (ANIA_06241)	74 kDa	20	0
2	40S ribosomal protein S10b (ANIA_03706)	18 kDa	2	0
3	Ubiquitin-protein ligase (Tom1) (ANIA_01966)	448 kDa	2	0
4	Telomere and ribosome associated protein Stm1, putative (ANIA_10614)	33 kDa	2	0
5	Uncharacterized protein (ANIA_01378)	22 kDa	3	0
6	RNP domain protein (ANIA_10276)	15 kDa	4	0

Fig S5.

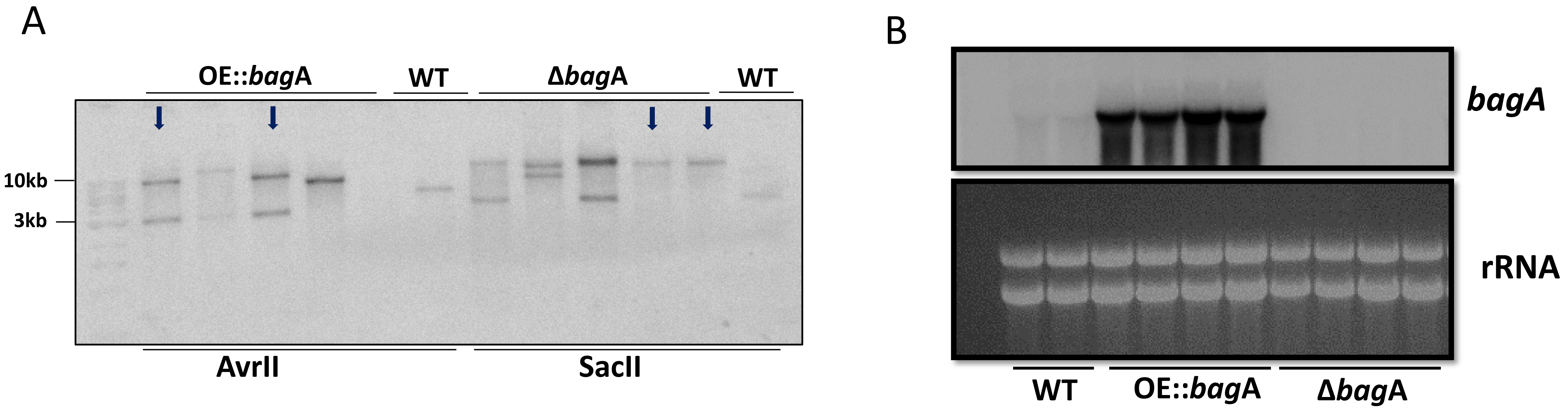
A
OE::bagA
WT
ΔbagA
WT
10kb
3kb
AvrII
SacII
B
bagA
rRNA
WT
OE::bagA
ΔbagA

Fig S6.

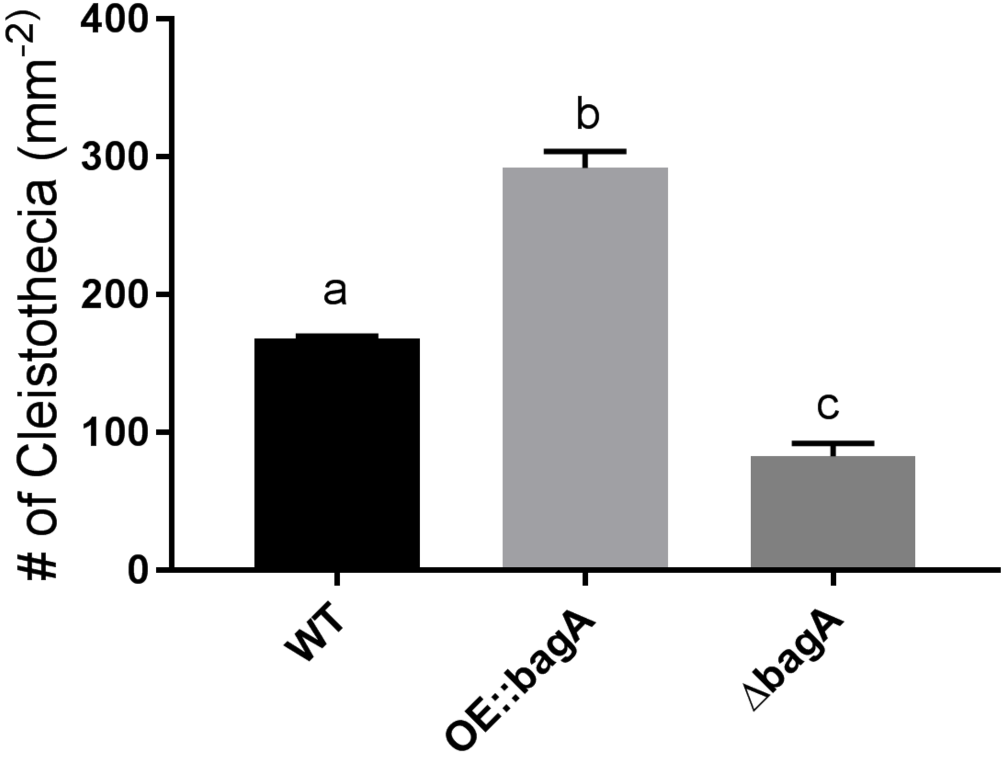

Fig S7.

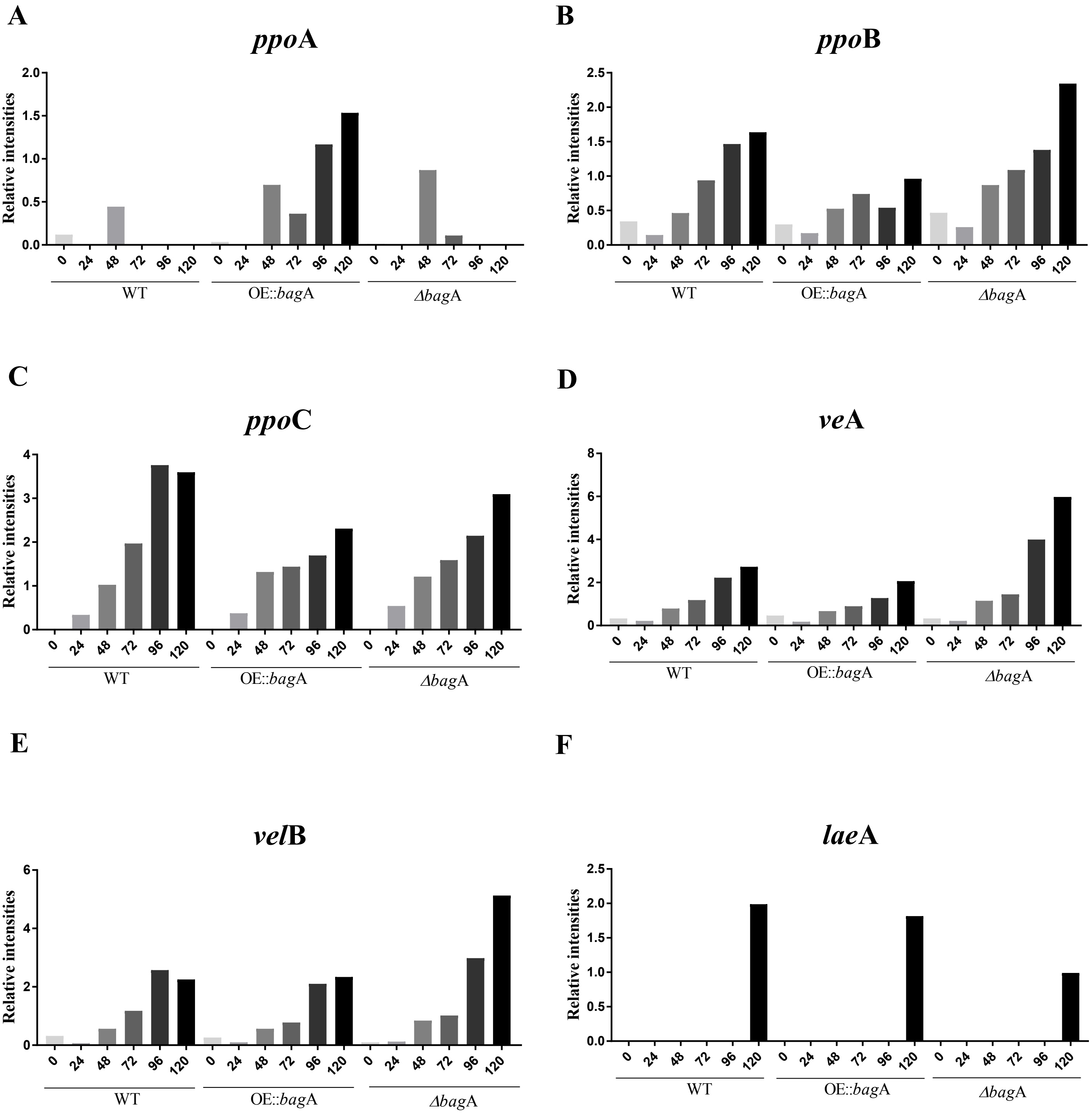
A
ppoA
B
ppoB
C
ppoC
D
veA
E
velB
F
laeA
Relative intensities
0
24
48
72
96
120
WT
OE::bagA
ΔbagA

Fig S8.

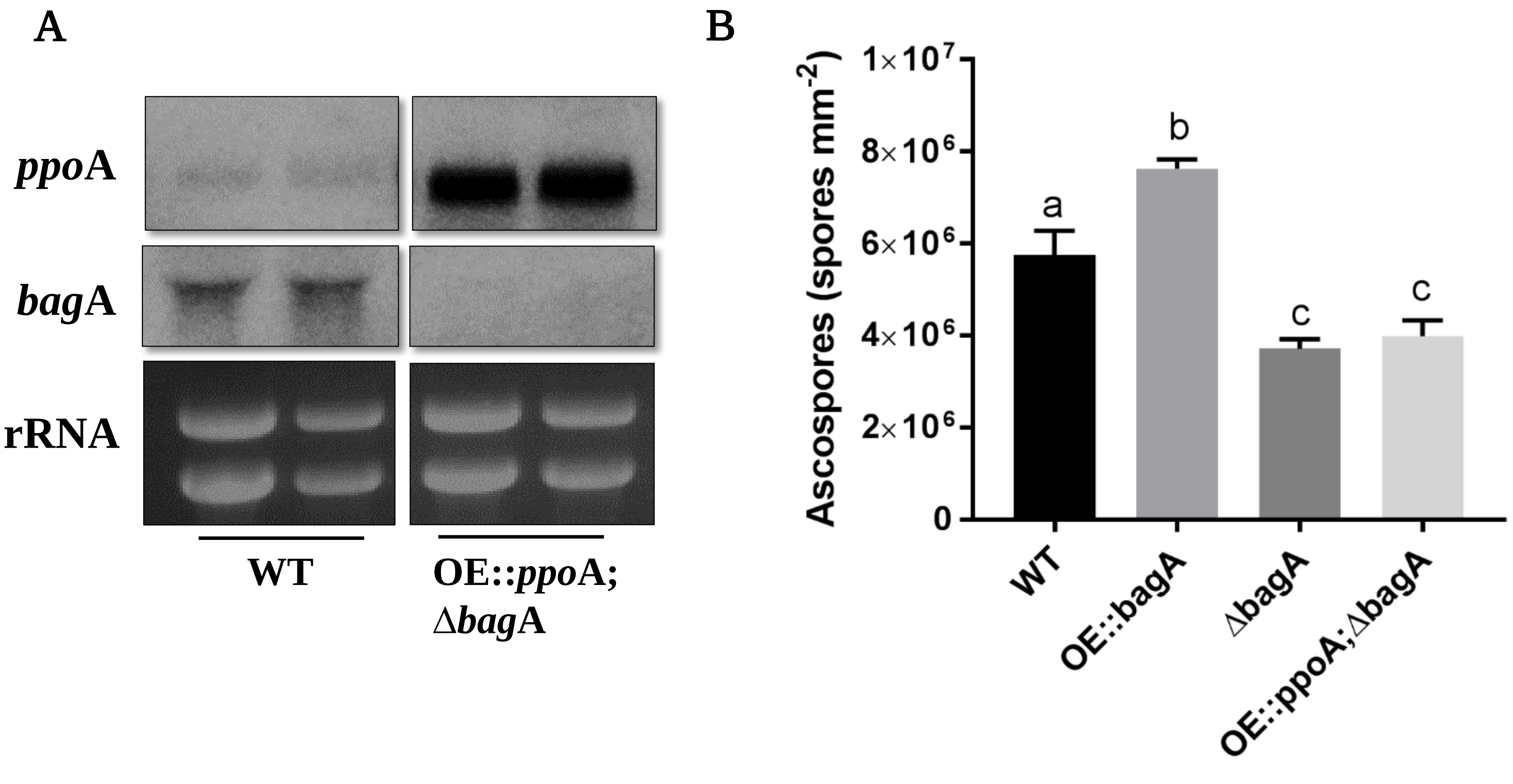
A
ppoA
bagA
rRNA
WT
OE::ppoA;
ΔbagA
B
Ascospores (spores mm-2)
1×10⁷
8×10⁶
6×10⁶
4×10⁶
2×10⁶
0
a
b
c
c
WT
OE::bagA
ΔbagA
OE::ppoA;ΔbagA

Fig. S9

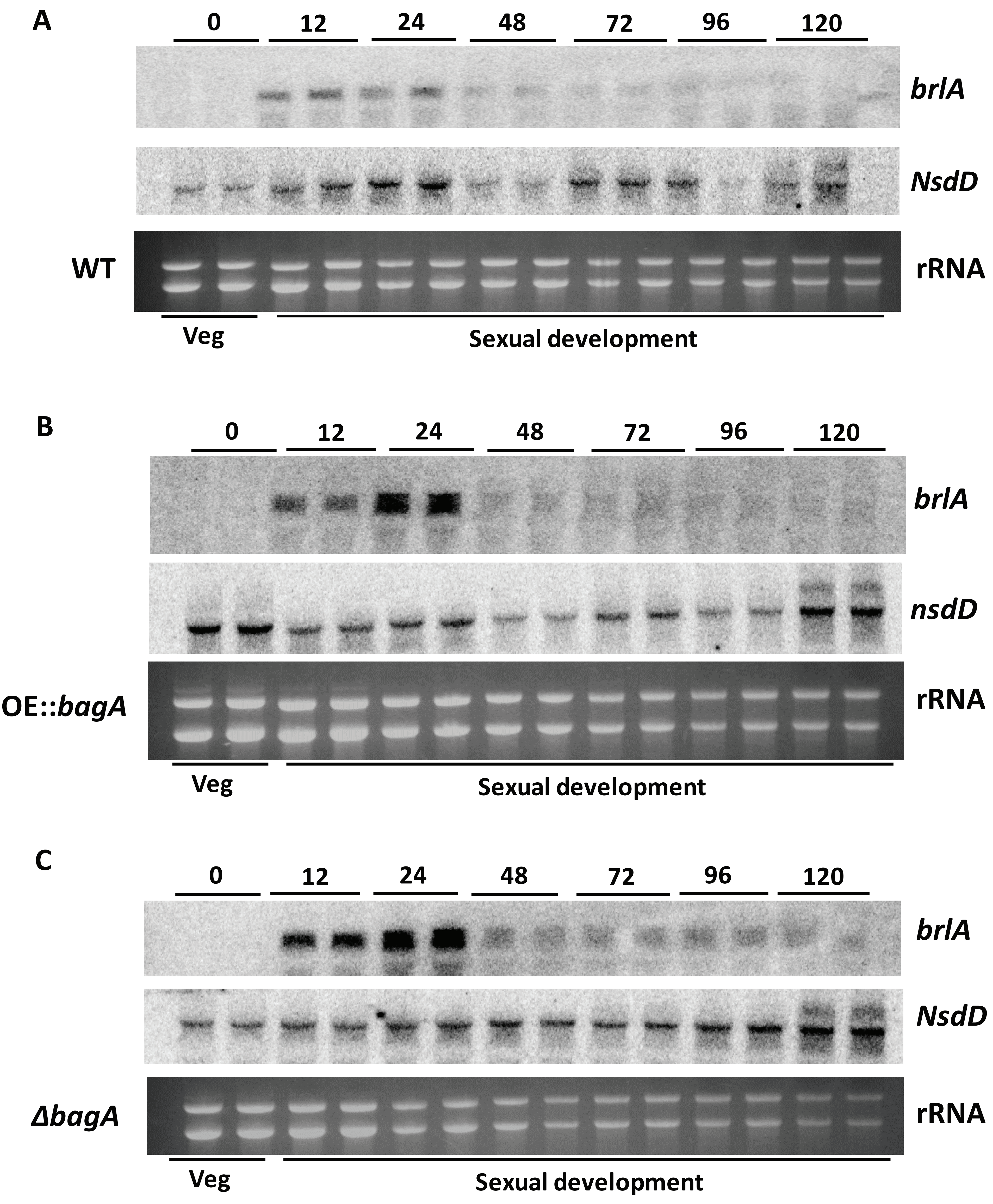
A
0
12
24
48
72
96
120
brlA
NsdD
WT
rRNA
Veg
Sexual development
B
0
12
24
48
72
96
120
brlA
nsdD
OE::bagA
rRNA
Veg
Sexual development
C
0
12
24
48
72
96
120
brlA
NsdD
ΔbagA
rRNA
Veg
Sexual development

Fig S10.

A

B

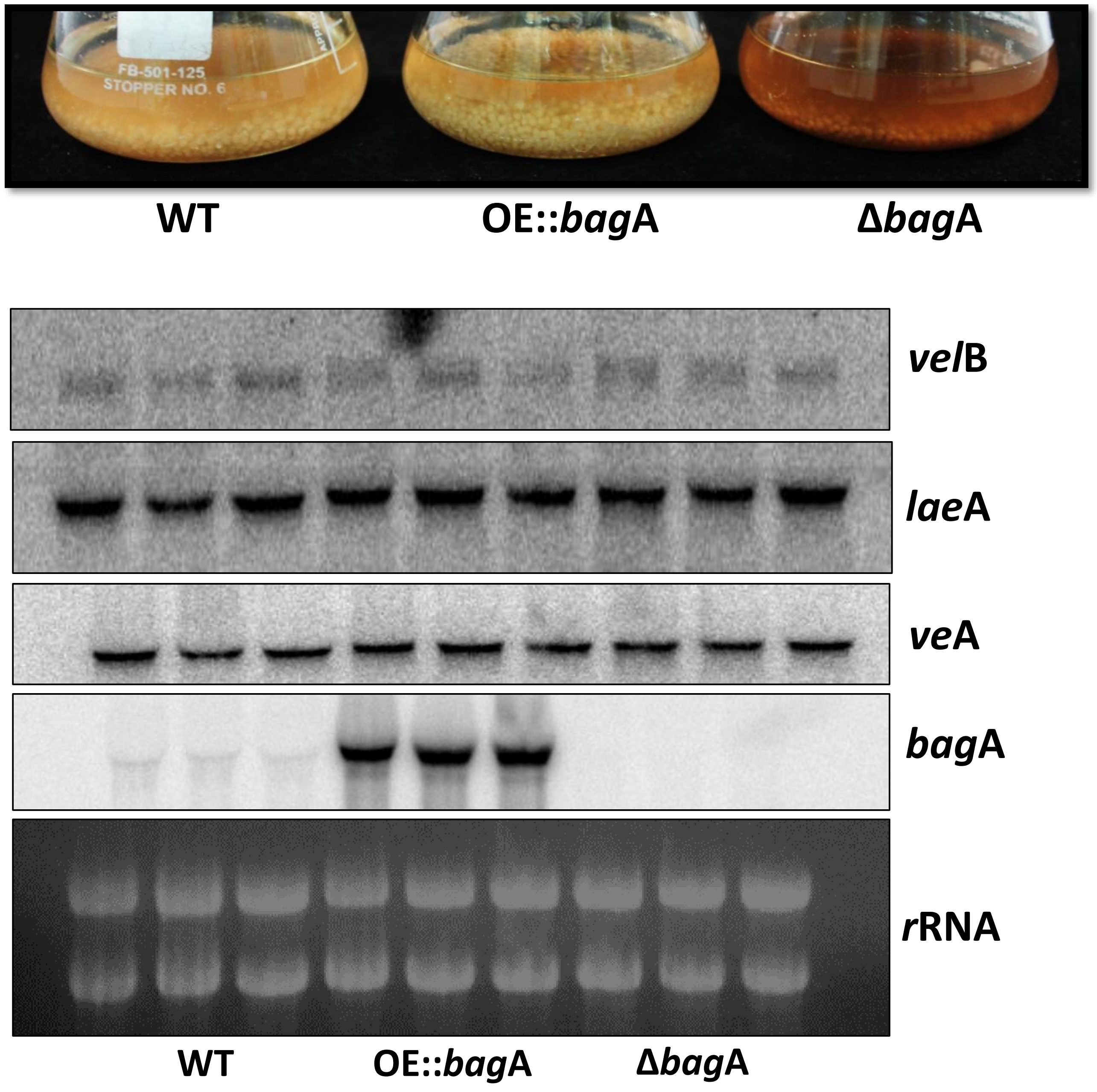
FB-501-125
STOPPER NO. 6
WT
OE::*bag*A
Δ*bag*A
*vel*B
*lae*A
*ve*A
*bag*A
*r*RNA
WT
OE::*bag*A
Δ*bag*A
