# Supplementary material for: A Bcl-2 Associated Athanogene (bagA) Modulates Sexual Development and Secondary Metabolism in the Filamentous Fungus Aspergillus nidulans
Source: Front Microbiol. 2018 Jun 15;9:1316. doi: 10.3389/fmicb.2018.01316 (PMC6013550; doi:10.3389/fmicb.2018.01316)
Supplement: Table S1 — List of strains used in this study. [file Table_1.pdf]

Table 1. List of strains used in this study.

Strain	Genotype	Source/Reference
RJMP103.5	Wild-type	PLoS Genet. 2013 Jan; 9(1): e1003193.
TNO2A7	*nku70::argB, riboB2, pyrG89, pyroA4, veA1*	Genetics. 2006 Mar; 172(3): 1557–1566.
RJMP101.19	*pyrG89; veA+*	PLoS Genet. 2013 Jan; 9(1): e1003193.
TSJA12.1	*nku70::argB, pyrG::gpdA(p)::bag*A*; riboB2, pyroA4, veA1*	This Study
TSJA13.1	*nku70::argB,* Δ*bag*A*:: pyrG; riboB2, pyroA4, veA1*	This Study
RSJA6.1	*pyrG89; pyrG:: gpdA(p)::bag*A	This Study
RSJA7.1	*pyrG89;* Δ*bag*A*:: pyrG*	This Study
RSJA11.1	*pyrG::gpdA(p)::bag*A*;* Δ*ppoA::metG*	This Study
RDIT5.8	*pyrG89*; *argB2*; *veA1*; *gpdA*(p)::*ppoA*::*trpC*	J Biol Chem. 2004 Mar 19;279(12):11344-53.
RSJA 14.1	*trpC::gpdA(p)::ppoA;* Δ*bagA::pyrG*	This Study
RSJA 18.1	*pyrG89; bag*A::Flag$_{3X}$:: *trpC*:: *pyrG*	This Study

Table 2. List of Primers used in this study

Name	Primer
SJ OE BAG Nid 5’	agt cgt gca aga aga gga t
SJ OEBAG Primer 1	atggatgagcaatgatggcaac
SJ OEBAG Primer 2	cca att cgc cct ata gtg agt cgt att acg gga ttt ata ata gac cag ccg g
SJ OEBAG Primer 3	cagctaccccgcttgagcagacatcaccatgaccaatctgctcccaca
SJ OEBAG Primer 4	caa cgc atc tgg tgt cac aat
SJ OEBAG Nid 3’ Rev	gaa gtc gcc gtt gca gct ct
SJ KOB Primer 2 Rev	cga tat caa gct atc gat acc tcg act cgg att tat aat aga cca gcc ggg
SJ KOB Primer 3 For	gtcgctgcagcctctccgattgtcgaatattaccttaaaccagttaaaatt
SJ Nid Bag int F	tcctcaccgtctgactgggcttc
SJ Nid Bag int R	gtc tcg tcg tcc aca act tca at
SJ 5' For Nid BAG EcoRI	aacggaattc atggatgagcaatgatggcaac
SJ 3' Rev Nid BAG SpeI	gagt actagt caacgcatctggtgtcacaat
SJ ppoC int F	actacaacccccgcaacctg
SJ ppoC int R	tggtcgtagtggcgtgtagg
SJ Nid Bag pyrG Rev	ggagatcgctcattgcgtgcggaaccgataagataggcgcagacc
SJ Bag 5' For	ttccgcacgcaatgagcgatctcc
SJ Bag Pyro Rev	ccttcgggcttcatagtggtcctgaaaacaatatattgggttatgt
SJ Mut D368 to S	caagtcatgattaaggcgtcgtccattgaagttgtggac
SJ ppoA int Nid F	gccgattcaccaaacctg
SJ ppoA int Nid R	cggatgagagatacgactg

SJ ppoB int F	acatccagccgagatctcg
SJ ppoB int R	cgc atg agg agc ttg tac at
SJ laeA int F	tcagcctccgaaccacccgaagaact
SJ laeA int R	ctccaggggccagcgatagacacgact
SJ velB int F	ttcgctagacagctcattctacg
SJ velB int R	tagtattcgttatccagaccatcg
SJ veA int F	ggaacgagggctcttatcgg
SJ veA int R	tgaagcagaagacatctgattgc
SJ Bag-Flag Rev	atcaccgtcatggtctttgtagtcggcctttgccaccgcatcgacctgat
SJ 3XFlag For	gactacaaagaccatgacggtgat
SJ trpC-pyrG For	taagcgcccactccacatctccacgagtcgaggtatcgatagcttgatat
SJ TrpC term Rev	gtggagatgtggagtgggcgctta
